# The Wnt5a Receptor, Receptor Tyrosine Kinase‐Like Orphan Receptor 2, Is a Predictive Cell Surface Marker of Human Mesenchymal Stem Cells with an Enhanced Capacity for Chondrogenic Differentiation

**DOI:** 10.1002/stem.2691

**Published:** 2017-08-30

**Authors:** Sally C. Dickinson, Catherine A. Sutton, Kyla Brady, Anna Salerno, Theoni Katopodi, Rhys L. Williams, Christopher C. West, Denis Evseenko, Ling Wu, Suzanna Pang, Roberta Ferro de Godoy, Allen E. Goodship, Bruno Péault, Ashley W. Blom, Wael Kafienah, Anthony P. Hollander

**Affiliations:** ^1^ Institute of Integrative Biology, University of Liverpool United Kingdom; ^2^ School of Cellular and Molecular Medicine, Faculty of Medical and Veterinary Sciences University of Bristol United Kingdom; ^3^ School of Clinical Sciences, Faculty of Medicine and Dentistry University of Bristol United Kingdom; ^4^ The University of Edinburgh, MRC Center for Regenerative Medicine Scotland United Kingdom; ^5^ Department of Orthopaedic Surgery University of Southern California (USC) Los Angeles California USA; ^6^ Department of Stem Cell Research and Regenerative Medicine University of Southern California (USC) Los Angeles California USA; ^7^ Royal National Orthopaedic Hospital Institute of Orthopaedics and Musculoskeletal Science, University College London Brockley Hill Stanmore United Kingdom; ^8^ The University of Edinburgh, Center for Cardiovascular Science Scotland United Kingdom; ^9^ David Geffen School of Medicine and Department of Orthopaedic Surgery Orthopaedic Hospital Research Center, University of California Los Angeles California USA

**Keywords:** Mesenchymal stem cells, Tissue engineering, Arthritis, Cellular therapy, Cell Signaling

## Abstract

Multipotent mesenchymal stem cells (MSCs) have enormous potential in tissue engineering and regenerative medicine. However, until now, their development for clinical use has been severely limited as they are a mixed population of cells with varying capacities for lineage differentiation and tissue formation. Here, we identify receptor tyrosine kinase‐like orphan receptor 2 (ROR2) as a cell surface marker expressed by those MSCs with an enhanced capacity for cartilage formation. We generated clonal human MSC populations with varying capacities for chondrogenesis. ROR2 was identified through screening for upregulated genes in the most chondrogenic clones. When isolated from uncloned populations, ROR2+ve MSCs were significantly more chondrogenic than either ROR2–ve or unfractionated MSCs. In a sheep cartilage‐repair model, they produced significantly more defect filling with no loss of cartilage quality compared with controls. ROR2+ve MSCs/perivascular cells were present in developing human cartilage, adult bone marrow, and adipose tissue. Their frequency in bone marrow was significantly lower in patients with osteoarthritis (OA) than in controls. However, after isolation of these cells and their initial expansion in vitro, there was greater ROR2 expression in the population derived from OA patients compared with controls. Furthermore, osteoarthritis‐derived MSCs were better able to form cartilage than MSCs from control patients in a tissue engineering assay. We conclude that MSCs expressing high levels of ROR2 provide a defined population capable of predictably enhanced cartilage production. Stem Cells *2017;35:2280–2291*

**Significance Statement**

Mesenchymal stem cells (MSCs) can be turned into cartilage‐forming cells. However, these stem cells vary from one donor to the other in their capacity to form cartilage, and they lose this capacity altogether if they are grown for too long in the laboratory. A marker protein on the surface of the stem cells might be used to predict which are best able to make cartilage. The authors generated clones of MSCs and showed that some of the clones are very good at making cartilage and some are very poor at doing so. Through comparison of these clones, a protein was identified, ROR2, that is present at higher levels on those MSCs that are very good at making cartilage. This new marker may help to ensure a more effective cell therapy for cartilage injuries.

## Dedication

This article is dedicated to the memory of Dr. Sally Dickinson, whose hard work and devotion to her research enabled the discoveries reported here.

## Introduction

A method of identifying and isolating mesenchymal stem cells (MSCs) with an enhanced capacity for cartilage formation should provide a useful tool in regenerative medicine. MSCs have been isolated from a number of anatomical locations in vivo and are believed to be of perivascular origin, where cells including pericytes are natural mesenchymal precursors 1, 2. Once cultured in vitro, MSCs are a heterogenous population defined by their capacity to adhere to tissue culture plastic and by their ability to differentiate into cartilage, bone, and fat lineages. Despite the absence of a definitive phenotypic marker, culture‐expanded MSCs have been defined as expressing CD105, CD73, CD44, and CD90 but lacking the expression of CD45, CD34, CD14, CD11b, CD79a, CD19, and HLA‐DR 3. Although the designation of MSCs as a true stem cell population is questionable 3, 4, 5, 6, their capacity to undergo chondrogenic and osteogenic differentiation 7, 8 is of clear therapeutic value. In particular, MSCs hold great promise in the regeneration of cartilage lesions. MSCs have been used to generate chondrocytes in vitro 8, 9, for the tissue engineering of cartilage 10, 11 and in numerous clinical studies. For example, one study has used autologous MSC‐generated chondrocytes to repair articular cartilage lesions in 40 patients with knee injuries 12. We engineered a 6‐cm human airway using autologous MSCs that was used successfully in the treatment of a patient with bronchial stenosis 13, 14. However, scale‐up of these procedures for the routine production of implantable cartilage of consistently high quality remains a significant challenge, in part because of the lack of standardized methods for isolation of a functional cell population optimized for chondrogenesis. A functional phenotypic marker for MSCs with enhanced chondrogenic potential would reduce variability between patients in autologous procedures and would also help to provide validation of the quality of MSC lines used for allogeneic therapies and exploration of the mechanisms of chondrogenesis. Here, we report the identification of inducible receptor tyrosine kinase‐like orphan receptor 2 (ROR2), the Wnt5a receptor, as a cell surface marker that is predictive of significantly enhanced chondrogenesis by MSCs, allowing production of 35% more cartilage and 95% higher tissue quality when used to repair cartilage lesions in sheep.

## Materials and Methods

### Antibodies and Primers

Details of specificity and suppliers of all antibodies are shown in Supporting Information Table S1. Details of all primers are shown in Supporting Information Table S2.

### Cell Types Used at Each Stage of Experimentation

Experimental work was undertaken in five stages using fluorescence‐activated cell sorting (FACS)‐sorted MSC clones, unfractionated (whole population) MSCs, FACS‐separated ROR2+ve, and ROR2–ve populations or fresh bone marrow, as described in Supporting Information Methods.

### Cell Culture

Iliac crest bone marrow aspirates were obtained from patients undergoing orthopedic surgery. All patients gave informed consent, and the study was performed in full accordance with local ethics guidelines (Southmead Research Ethics Committee Ref 78/01). Bone marrow was added into tissue culture flasks containing expansion medium consisting of Dulbecco's modified Eagle's medium supplemented with 1,000 mg/l glucose, 10% fetal bovine serum from a selected batch (Thermo Scientific Hyclone, Loughborough, UK, http://www.thermofisher.com), 100 units/ml penicillin, 100 μg/ml streptomycin (all from Sigma, Poole, UK, http://www.sigmaaldrich.com), and 2 mM Glutamax‐I (Invitrogen Ltd, Paisley, UK, http://www.thermofisher.com). Non‐adherent cells were removed during medium changes and adherent MSCs were proliferated in the presence of 2 ng/ml fibroblast growth factor‐2 (FGF‐2; PeproTech, London, UK, http://www.peprotech.com). MSCs were passaged when 80% confluent and preserved in liquid nitrogen until further use. After thawing, cells were proliferated further in the presence of FGF‐2 and the number of population doublings (PDs) was calculated at each passage. Cellular senescence was monitored regularly by staining for β‐galactosidase activity (Senescence Cells Histochemical Staining Kit; Sigma).

### Sheep Model for Testing the In Vivo Chondrogenic Capacity of MSCs

Full details of all operative procedures are described in Supporting Information Methods. Isolated inducible ROR2 (iROR2)+ve cells, ROR2–ve cells, or unfractionated whole population cells from eight human donors were seeded onto polyglycolic acid (PGA) scaffolds and used to engineer cartilage for 35 days as described earlier. Bone marrow aspirates were obtained from eight skeletally mature (over 4 years old) female English Mule sheep (average weight 75.75 ± 7.34 kg) and plastic‐adherent ovine MSCs were obtained by iliac crest biopsy and proliferated using the same methods and reagents as described above for human cells. The ovine MSCs were seeded dropwise at 1 × 10^6^ cells per square centimeter onto Avitene Ultrafoam collagen sponges (3 mm thickness; Bard, Crawley, UK, http://www.barduk.com) and incubated overnight at 37°C (Cell Bandage 15). A 6‐mm diameter chondral defect was prepared in the left medial femoral condyle of each of the sheep and lined with the Cell Bandage before the human tissue engineered cartilage was added to the defect and sutured in place. The implants remained in vivo for 3 months before the sheep were killed. All animal procedures were approved by the Royal Veterinary College Ethics committee and carried out under a project licence granted by the UK Home Office (PPL 70/6964), in accordance with the Animals (Scientific Procedures) Act of 1986.

Detailed methods are included in Supporting Information online data.

## Results

### Production of MSC Clonal Lines with Varying Capacity for Chondrogenesis

**Figure 1:**
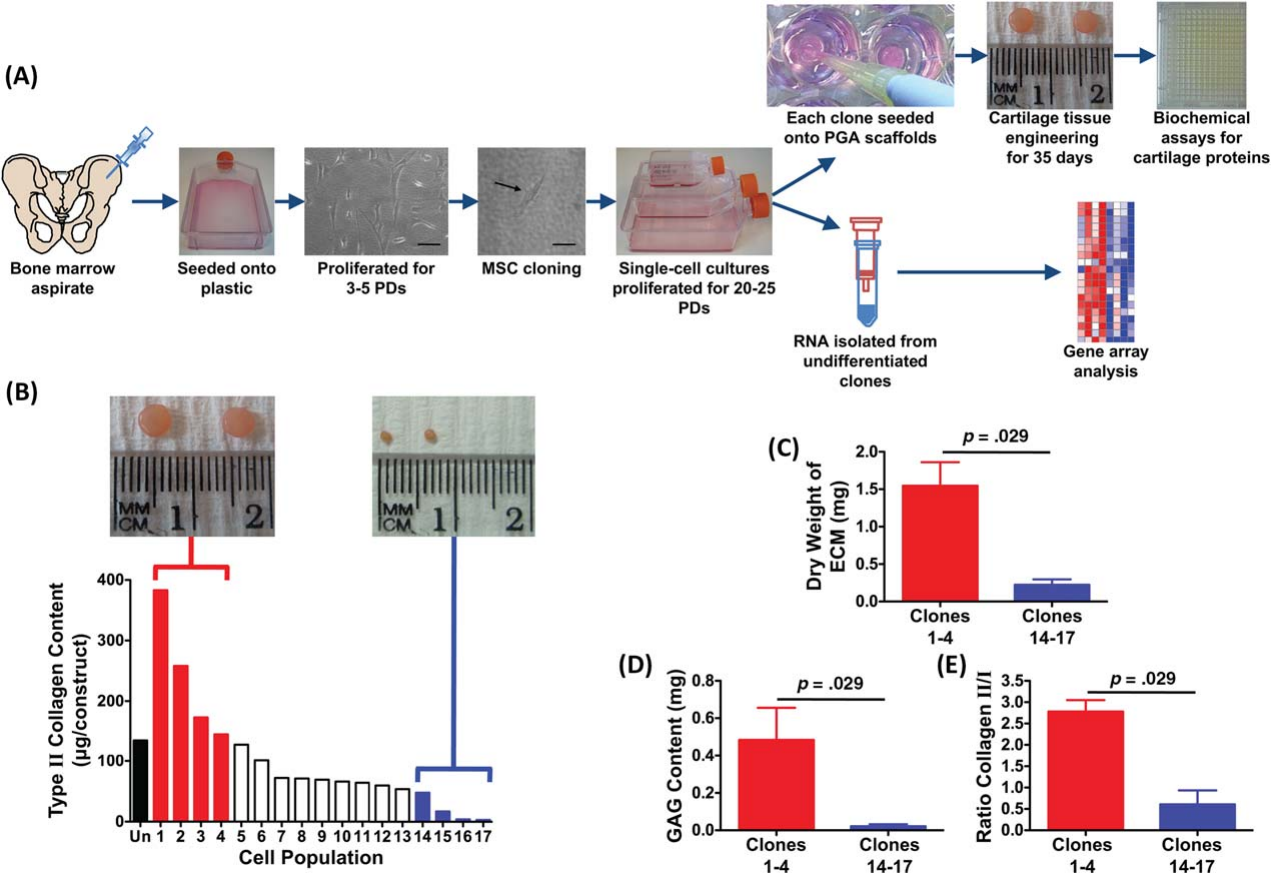
Clonal analysis of human bone marrow mesenchymal stem cells (MSCs). **(A):** A scheme of the approach to isolating MSC clones and analyzing each one for chondrogenic capacity and selected clones for mRNA expression by gene array. MSC isolation was by plastic adhesion and proliferation through a number of population doublings with FGF‐2, cloning was by unbiased flow cytometry sorting with no selecting antibody, chondrogenic capacity was by tissue engineering for 30 days on polyglycolic acid scaffolds and gene expression was by Affymetrix gene array (size bars = 100 μm). **(B):** Chondrogenesis measured as the total type II collagen content by enzyme linked immunosorbent assay, of tissue engineered cartilage (*n* = 1) made from unfractionated MSCs or MSC clones (1–17). Insets show the typical appearance of tissue engineered cartilage. Cartilage engineered from the four most chondrogenic clones (1–4, red bars) and the four least chondrogenic clones (14–17, blue bars) was further analyzed for dry weight of extracellular matrix formed **(C)**, content of proteoglycan measured as GAG **(D)**, and the ratio of type II to type I collagen **(E)**. Each scale bar is the mean ± SEM of *n* = 4 clones. Comparison of differences between groups was by two‐tailed Mann‐Whitney *U* test. Abbreviations: ECM, extracellular matrix; PD, population doubling; PGA, polyglycolic acid.

Our strategy for identifying a cell surface marker, summarized in Figure 1A, was to clone human MSCs by using a flow cytometer to deposit single bone marrow‐derived MSCs into individual wells of 96‐well plates, without any antibody‐based selection. The cells were then expanded through multiple PDs, providing a large enough number of cells of each clone for screening by cartilage tissue engineering and by subsequent gene array analysis of individual undifferentiated clones. Each clone was cultured in expansion medium containing 2 ng/ml FGF‐2, which supports the proliferation of single‐cell‐derived populations 16 and maintains chondrogenic differentiation potential 17. The single cells began to divide after 2–3 days and those clones that continued to proliferate reached 90% confluence in 13–22 days. They were then passaged and re‐seeded into 12‐well plates and subsequently 25, 75, and 175 cm^2^ flasks, as required, as they continued to expand in number. The clones were prepared from MSCs from six different patients, with varying cloning efficiency, as shown in Supporting Information Table S3. Approximately 1 × 10^6^ cells were needed to assess multilineage differentiation and therefore each clone was required to undergo a minimum of 20 PDs for further analysis. The amount of cellular senescence in the clonal populations was estimated by staining for β‐galactosidase activity and those clones showing any evidence of senescence were excluded from further investigation. Only clones derived from one of the patient bone marrow samples, PN5, were used for tissue engineering and gene array analysis. We opted for use of clones from a single patient because of the observation that this sample generated the majority of clones and by excluding other samples we could reduce variability in the screening procedure.

We analyzed 17 of the stable clones from PN5 and found each one to have a unique capacity for cartilage formation, as judged by type II collagen content of the engineered tissue measured using an epitope‐specific enzyme linked immunosorbent assay. The type II collagen content ranged from 1 to 383 µg per tissue engineered construct (Supporting Information Table S4). There was a significant inverse correlation between the time taken for the clones to undergo 20 PDs and the chondrogenic potency as judged by the type II collagen content of tissue engineered cartilage (Supporting Information Fig. S1). The same clones were tested for their in vitro osteogenic and adipogenic potential. As for chondrogenesis, there was a wide variation in the differentiation capacity for these two pathways (Supporting Information Fig. S2); however, the degree of osteogenesis or adipogenesis did not correlate with the chondrogenic capacity (Supporting Information Table S4).

We selected the four clones with the greatest capacity to form cartilage (1–4) and the four clones with the poorest capacity to form cartilage (14–17) based on their capacity for type II collagen production (Fig. 1B) and further analyzed their chondrogenic potential. Clones 1–4 were found to generate cartilage with a significantly higher dry weight (Fig. 1C), proteoglycan content (Fig. 1D), and collagen II/I ratio (Fig. 1E), than clones 14–17. This analysis, therefore, validated our classification of clones 1–4 as highly chondrogenic and clones 14–17 as poorly chondrogenic, enabling their use in screening studies.

### Identification of a Marker of Selected Clonal Lines by Gene Array Analysis

**Figure 2:**
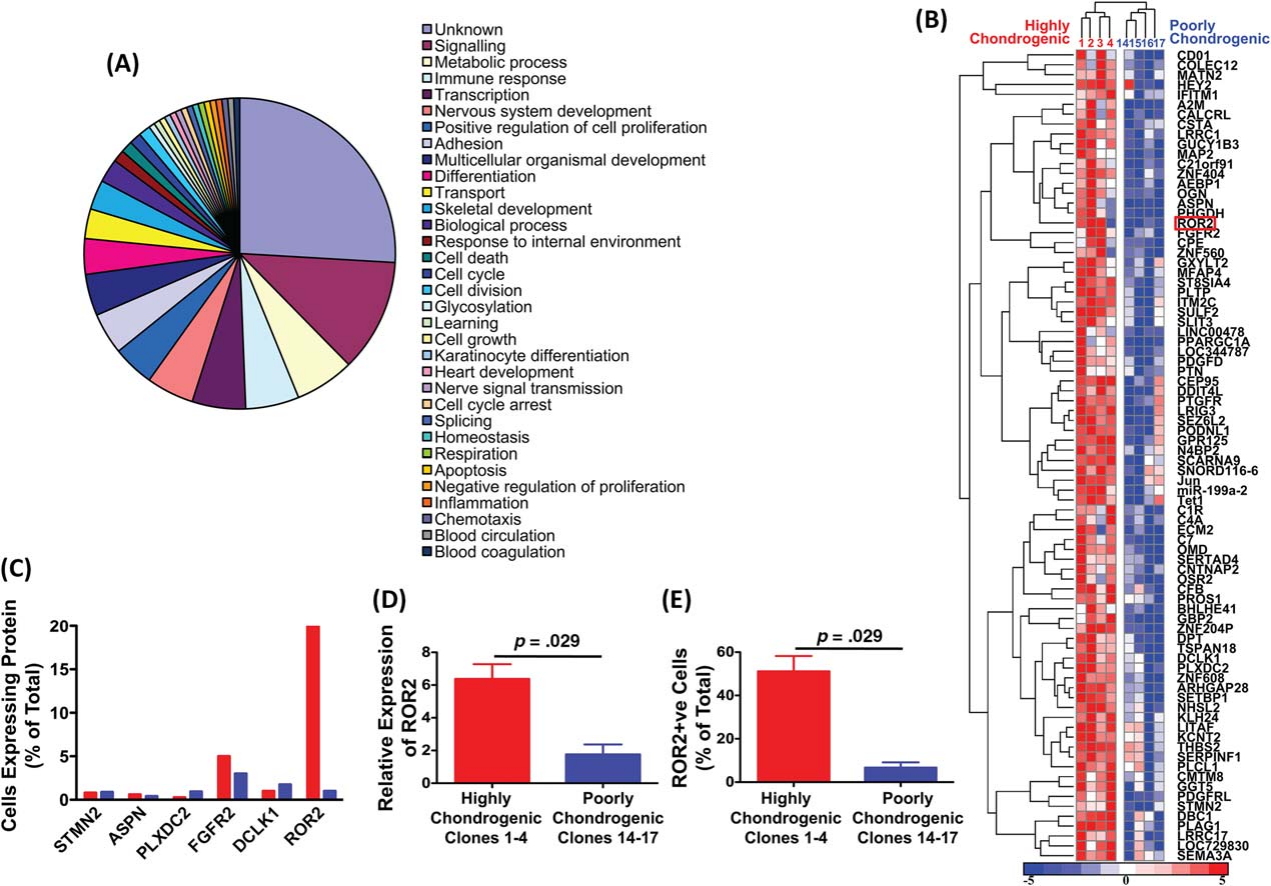
Identification of cell surface markers upregulated on highly chondrogenic mesenchymal stem cell clones. **(A):** Ontology of genes significantly upregulated on highly chondrogenic clones. The chart shows the function of those genes which are at least 1.3‐fold upregulated on the four most highly chondrogenic clones (clones 1–4) in comparison with the four least chondrogenic clones (clones 14–17). Only genes showing a significant change in *p* < .05 are included (two‐way analysis of variance). **(B):** Heatmap and hierarchical clustering of the 82 genes that were significantly upregulated on the highly chondrogenic clones. Each column is one clone and each row is one gene. **(C):** Flow cytometry analysis of the percentage of cells expressing each of six proteins identified from the gene array analysis and known to contain membrane‐spanning domains. Red bars are results for one highly chondrogenic clone (clone 1) and blue bars are results for one poorly chondrogenic clone (clone 15). The proteins analyzed were stathmin‐like 2 (STMN2), asporin (ASPN), plexin domain containing 2 (PLXDC2), fibroblast growth factor receptor 2 (FGFR2), doublecortin‐like kinase 1 (DCLK1), and receptor tyrosine kinase‐like orphan receptor 2 (ROR2). **(D):** Real‐time quantitative polymerase chain reaction analysis of ROR2 gene expression and **(E)** flow cytometry analysis of ROR2 protein expression by the four highly chondrogenic clones 1–4 (red bars) and four poorly chondrogenic clones 14–17 (blue bars). Each bar is the mean ± SEM of *n* = 4 clones. Comparison of differences between groups was by two‐tailed Mann‐Whitney *U* test. Abbreviation: ROR2, receptor tyrosine kinase‐like orphan receptor 2.

We isolated the mRNA of undifferentiated cells from the highly and poorly chondrogenic clones that had been expanded with FGF‐2 and serum to prime them for chondrogenic differentiation, but not yet induced to differentiate with transforming growth factor (TGF)‐β3. We went on to investigate differential gene expression between the two groups, to identify genes predictive of enhanced chondrogenic potential upon subsequent differentiation. Ontological analysis of the function of those genes differentially upregulated by the highly chondrogenic clones indicated that the majority of those with a known function were cell signaling genes (Fig. 2A; Supporting Information Table S5). This is important as cell signaling pathways play a critical role in determining the differentiation fate of MSCs. Heat map analysis of the genes that were upregulated on highly chondrogenic clones 1–4 compared with poorly chondrogenic clones 14–17 confirmed that there was clustering of upregulated genes between these two groups (Fig. 2B). The 82 genes shown in this heat map are listed in Supporting Information Table S6 in order of statistical significance of the differential expression between clone groups. As the purpose of this study was to identify cell surface markers predictive of chondrogenesis, we selected a subset of 24 of the differentially expressed genes that contained at least one membrane‐spanning domain (Supporting Information Table S7). Only one of the genes, *ROR2*, showed a clear differential protein expression upon preliminary analysis by flow cytometry (Fig. 2C). Differential upregulation of ROR2 mRNA and protein was confirmed as significant (four highly chondrogenic clones compared with four poorly chondrogenic clones) by real time quantitative polymerase chain reaction (Fig. 2D) and flow cytometry (Fig. 2E), respectively.

### Induction of ROR2 Expression by Chondrogenic MSCs

**Figure 3:**
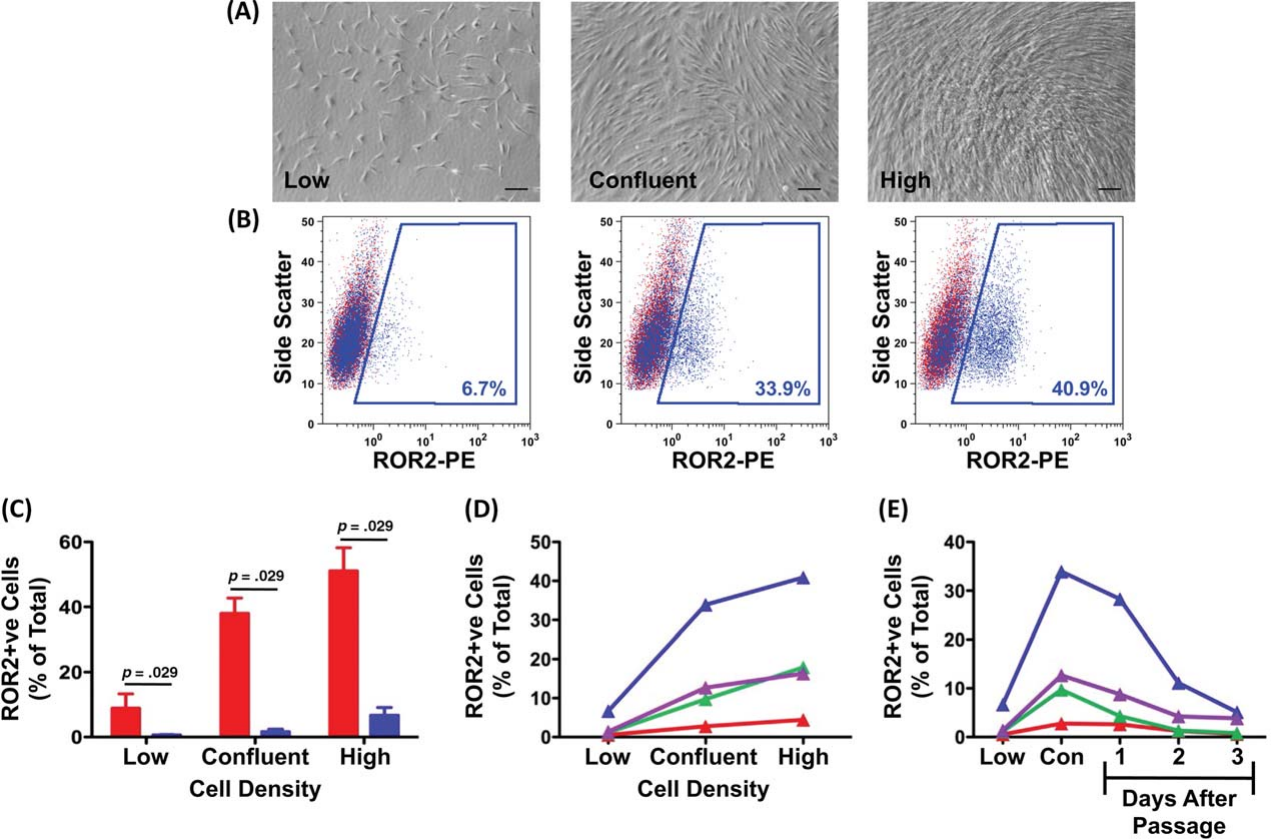
Upregulation of receptor tyrosine kinase‐like orphan receptor 2 (ROR2) protein expression by increased cell density. Mesenchymal stem cells (MSCs) were seeded into tissue culture flasks and harvested for analysis of ROR2 expression by flow cytometry after growing to low density (3–6 days), confluence (7–14 days), or high density (14–18 days). **(A):** The typical appearance of MSCs at each density range. Scale bar = 100 μm. **(B):** Detection of ROR2 by flow cytometry in a representative sample of cells at each density range. Events in red are cells labeled with isotype control antibody and events in blue are cells labeled with anti‐ROR2 antibody and detected using PE fluorescence. **(C):** Analysis of ROR2 expression at different cell densities on highly chondrogenic clones 1–4 (red bars) and poorly chondrogenic clones 14–17 (blue bars). Each bar is the mean ± SEM of *n* = 4 clones. Comparison of differences between groups was by two‐tailed Mann‐Whitney *U* test. **(D):** Analysis of ROR2 expression at different cell densities on unfractionated MSCs from four different donors without passage of the cells during the experiment. Each line represents one donor. **(E):** Analysis of ROR2 expression at different cell densities on unfractionated MSCs from four different donors before and after passage. Each line represents one donor. Abbreviations: Con, confluent; ROR2, receptor tyrosine kinase‐like orphan receptor 2; PE, phycoerythrin.

Initial observations indicated higher *ROR2* gene expression in when MSCs were cultured at a high cell density. As illustrated in Figure 3A, undifferentiated MSC clones were grown to low density, where cell‐to‐cell contact was minimal (mean ± SEM of 7,137 ± 584 cells per square centimeter), to confluence (38,083 ± 6,515 cells per square centimeter) or to high density, where 100% confluent cells were maintained in culture for an additional 6 days (46,417 ± 7,494 cells per square centimeter). ROR2 protein expression was then determined by flow cytometry for cells at each density (Fig. 3B). For highly chondrogenic clones 1–4, we observed a clear increase in ROR2 expression with increasing cell density, whereas there was minimal expression of ROR2 on the poorly chondrogenic clones even in high density culture (Fig. 3C). These data demonstrate that the highly chondrogenic clones can be induced to express ROR2. For the remainder of this article, we have, therefore, adopted the terminology of iROR2+ve cells to describe cultured cells showing an increased ROR2 expression, induced through increasing cell density in culture. MSCs which do not show any increase in ROR2 during culture expansion are described as ROR2 negative (ROR2–ve) cells. ROR2 expression on uncultured MSCs is referred to as percentage ROR2 expression relative to another cell surface marker.

We went on to investigate ROR2 expression by unfractionated/uncloned MSCs from four different patients. There was a clear increase in ROR2 expression with increasing cell density for all four patients, although there was also marked variation in the extent of expression between the different patients (Fig. 3D). When these cells were passaged and replated at low density, there was a marked fall in ROR2 expression over the first 3 days of the new passage (Fig. 3E). Therefore, for all subsequent studies, ROR2 expression was measured only after the cells had been grown to high density, allowing comparison to be made between different subsets of cells.

### Isolation and Characterisation of iROR2+Ve and ROR2–ve MSCs

**Figure 4:**
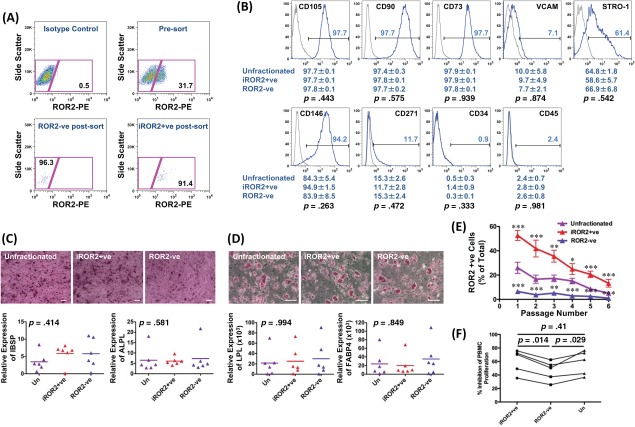
Characterization of inducible receptor tyrosine kinase‐like orphan receptor 2 (iROR2)+ve and ROR2–ve mesenchymal stem cell (MSC) populations. **(A):** MSCs were grown to high density and the iROR2+ve and ROR2–ve populations were isolated using sterile flow cytometry. **(B):** Sorted or unfractionated MSCs were analyzed by flow cytometry for the expression of marker proteins. Population frequencies for each marker are shown below the plots (mean ± SEM; *n* = 6). There was no statistically significant difference in any of the marker proteins (Kruskal‐Wallis). **(C):** Osteogenic differentiation of the sorted and unfractionated (Un) populations from *n* = 6 donors shown by alizarin red staining (size bars = 100 μm) and by real‐time quantitative polymerase chain reaction (qPCR) for integrin‐binding sialoprotein (bone sialoprotein) and alkaline phosphatase. There were no significant differences in gene expression (Kruskal‐Wallis). **(D):** Adipogenic differentiation of the sorted and unfractionated (Un) populations from *n* = 6 donors shown by oil red O staining (scale bars = 100 μm) and by real‐time qPCR for lipoprotein lipase and fatty acid binding protein 4. There were no significant differences in gene expression (Kruskal‐Wallis). **(E):** Maintenance of the iROR2+ve and –ve phenotypes was tested for six passages after sorting. Each point is the mean ± SEM; *n* = 6 for each population). *, *p* = 0.2000; **, *p* = 0.0294; ***, *p* = 0.0286 versus the unfractionated whole population, by two‐tailed Mann‐Whitney *U* test. **(F):** Suppression of lymphocyte proliferation by MSCs. The percentage inhibition of lymphocyte proliferation was measured after coculture of the mononuclear cells with sorted or unfractionated (Un) MSCs from *n* = 5 donors. Statistical analysis was by paired *t* test. Abbreviations: FABP4, fatty acid binding protein 4; IBSP, integrin‐binding sialoprotein; LPL, lipoprotein lipase; PE, phycoerythrin; ROR2, receptor tyrosine kinase‐like orphan receptor 2.

To study further, the iROR2+ve MSCs identified in the unfractionated population, it was essential to isolate them, as well as ROR2–ve MSCs, as separate populations. We were able to isolate iROR2+ve and ROR2–ve MSCs from confluent cultures of the unfractionated cells by labeling with an anti‐ROR2 antibody and using sterile cell sorting to collect the different populations. (Fig. 4A). The experiment was repeated using MSCs from *n* = 8 donors with a final purity (mean ± SEM) of 97.3% ± 0.7% for ROR2–ve and 90.2% ± 0.4% for iROR2+ve MSCs.

While we hypothesize that the iROR2+ve MSCs will be more highly chondrogenic than iROR2–ve MSCs, it is important to determine whether both of these populations have retained the characteristics of MSCs, namely expression of established cell surface markers, multidifferentiation capacity and prolonged proliferative life‐span. We, therefore, started our analyzes by testing each of these stem cell attributions before going on to determine the chondrogenic potential of these MSCs. A range of cell surface markers have been used to define the unfractionated MSC population 3, and we, therefore, considered it important to know if iROR2 expression was associated with either an increase or a decrease in expression of these known markers. We, therefore, determined levels of nine different markers by flow cytometry, comparing iROR2+ve MSCs with ROR2–ve and unfractionated MSCs. There were no significant differences in the expression of CD105, CD90, CD73, VCAM1, STRO‐1, CD146, CD271, CD34, and CD45 between the different MSC populations (Fig. 4B), demonstrating that ROR2 is an independent marker, not directly associated with any of the known markers currently used to define this type of stem cell. The iROR2+ve, ROR2–ve, and unfractionated populations of MSCs were all multipotential progenitor cells as judged by their capacity to differentiate to more than one lineage and there was no apparent difference between the populations in their capacity to undergo osteogenesis (Fig. 4C) or adipogenesis (Fig. 4D). These data indicate that the iROR2+ve and ROR2–ve cells have each retained their MSC properties and any differences observed in chondrogenic capacity must, therefore, be a result of differences in stem cell potential rather than stage of differentiation of the cell subsets.

If ROR2 selection is to be of practical use, it is important to determine whether the isolated iROR2+ve cells retain a higher expression of this marker after multiple PDs. Figure 4E shows that over six passages, the isolated ROR2–ve population remained negative throughout, never recovering the capacity to upregulate ROR2. The iROR2+ve and unfractionated populations both gradually lost expression of iROR2 with increasing passage number; however, the iROR2+ve fraction retained a significantly raised expression of ROR2 compared with unfractionated cells at all passages. The fall in ROR2 expression with increasing passage is coincident with a reduced capacity for chondrogenesis. This is illustrated by the macroscopic appearance of tissue engineered cartilage made from early and late passage MSCs (Supporting Information Fig. S3A, S3B) and the decreased content of cartilage specific molecules at late compared with early passages (Supporting Information Fig. S3C, S3D). Nevertheless, the consistently raised level of iROR2 compared with controls throughout this extended culture period indicates that the phenotype is relatively stable and that the benefit of selection may be maintained as the cells are expanded.

MSCs have been previously shown to be immunoregulatory, suppressing third party T‐cell proliferation 18, 19, 20. We, therefore, compared iROR2+ve MSCs with ROR2–ve and unfractionated MSCs for their capacity to inhibit T‐cell proliferation in vitro. This important property of MSCs was no different in iROR2+ve and unfractionated MSCs; however, for each of the five patients studied, ROR2–ve MSCs had a lower capacity to inhibit T‐cell proliferation compared with iROR2+ve or unfractionated populations and this difference was significant (Fig. 4F).

### Functional Advantage of iROR2+ve in Cartilage Tissue Engineering In Vitro

**Figure 5:**
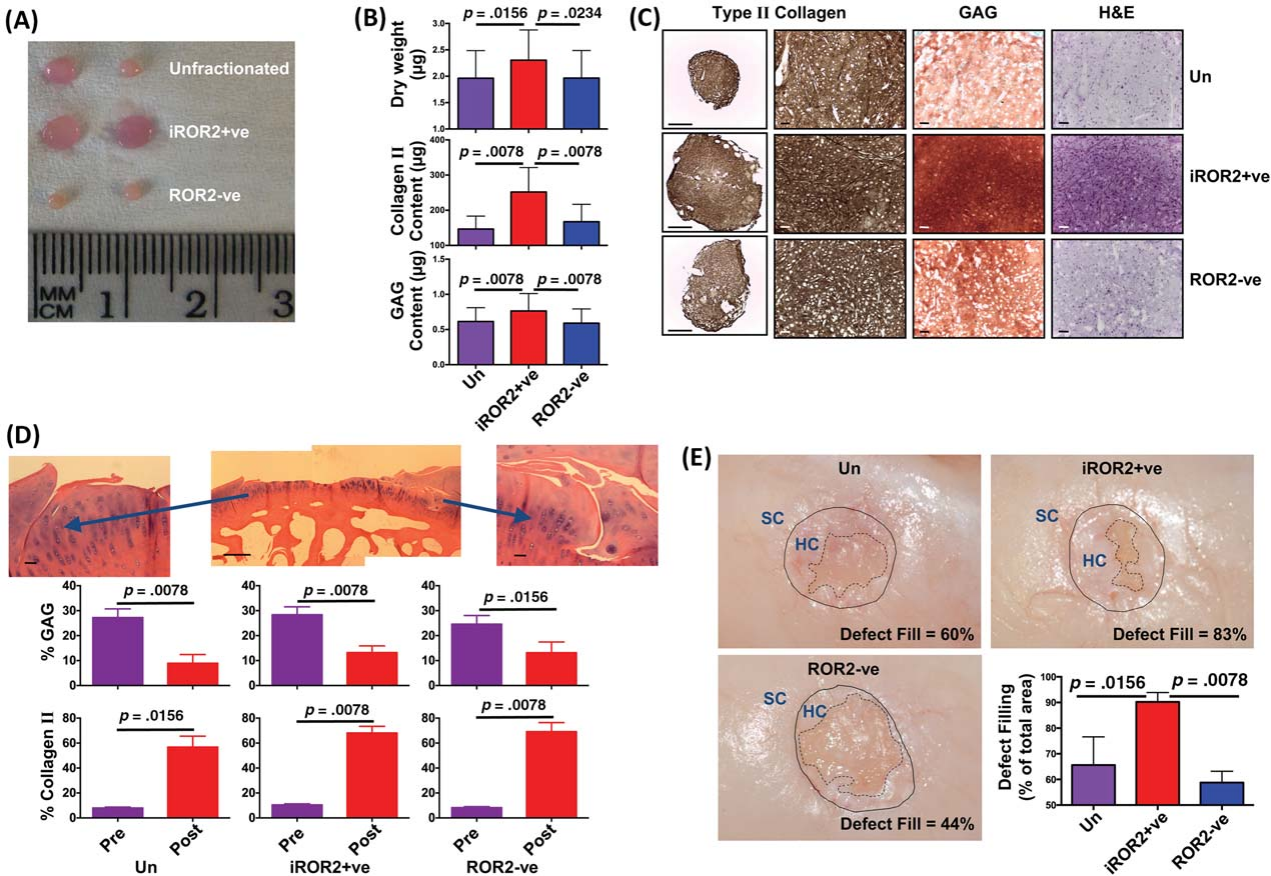
Enhanced chondrogenic capacity of inducible receptor tyrosine kinase‐like orphan receptor 2+ve mesenchymal stem cell (MSC). Sorted or unfractionated human MSCs were used for cartilage tissue engineering and analyzed after 35 days in vitro **(A–C)** or after 3 months in vivo in a sheep model **(D, E)**. In (A), the typical macroscopic appearance is shown for duplicate cartilage constructs engineered from unfractionated or sorted MSCs in vitro. (B): The biochemical content of tissue engineered cartilage (mean ± SEM; *n* = 8). Statistical analysis by Wilcoxon matched pairs test. (C): Low‐magnification (column 1, scale bar = 1,000 μm) and high‐magnification (columns 2–4, size bar = 100 μm) histological images stained for type II collagen, with safranin‐O for proteoglycan (GAG) or with H&E. (D): Cartilage engineered from sorted or unfractionated MSCs from eight human donors were implanted into defects within the femoral condyles of sheep stifle joints without immunosuppression. Upper panels show safranin‐O staining (scale bar = 100 μm). The percentage proteoglycan content (measured as GAG) and percentage Type II collagen content was measured immediately before implantation (purple bars) or 3 months after implantation (red bars). Statistical analysis by Wilcoxon matched pairs test. (E): Defect filling in the sheep cartilage repair model after 3 months. The in situ appearance of representative samples is shown, with the integration site between the natural sheep cartilage and the implanted human cartilage indicated. The defect filling was measured for each sample and expressed as a percentage of total area (*n* = 8 for each experimental group). Statistical analysis was by Wilcoxon matched pairs test. Abbreviations: HC, human cartilage; iROR2, inducible ROR2; ROR2, receptor tyrosine kinase‐like orphan receptor 2; SC, sheep cartilage; Un, unfractionated.

MSCs were isolated from eight patients and then either fractionated into the iROR2+ve and ROR2–ve populations or cultured without fractionation as a control. The three subpopulations from each donor were each seeded onto polyglycolic acid scaffolds and cartilage tissue engineering was induced using a combination of TGFβ3, ascorbic acid, and insulin. Typical examples of the macroscopic appearance of cartilage after 35 days in vitro are shown in Figure 5A, illustrating a tendency for larger amounts of tissue to be formed by the iROR2+ve cells. This observation is confirmed by the significantly higher dry weights of cartilage constructs engineered using iROR2+ve MSCs compared with either ROR2–ve MSCs or unfractionated controls and by the detailed biochemical analysis of the tissue engineered cartilage from each MSC sub‐population from all 8 donors (Fig. 5B). Whether comparing iROR2+ve cells with unfractionated MSCs or with the ROR2–ve fraction, there was a significantly higher content of both type II collagen and proteoglycan. Low and high power immunolocalization for collagen II and histological staining for proteoglycans or extracellular matrix (ECM) also shows a more extensive cartilage formation when using iROR2+ve cells to engineer cartilage (Fig. 5C).

### Therapeutic Advantage of iROR2+ve over Unfractionated MSCs in a Sheep Cartilage Repair Model

We established a functionally loaded in vivo model for the implantation of human tissue engineered cartilage into full thickness articular cartilage lesions in the ovine medial femoral condyle without the use of immunosuppressive drugs. Immune rejection was avoided in this model because we implanted a “Cell Bandage” formed from sheep undifferentiated MSCs seeded onto a collagen sponge at the interface between host and implant (Supporting Information Fig. S4). We designed the Cell Bandage as a mechanism for driving integration of cartilage 15, 21; however, the undifferentiated MSCs are also immunoregulatory 22 and so will create a zone of tolerance at the interface site. Additional protection from immune rejection may have resulted from growing the engineered cartilage in vitro for 35 days, allowing a dense ECM to become established before implantation, creating an immune privileged site through physical inhibition of the migration of elements of the immune system. In preliminary experiments using unfractionated cells, we observed through histological analysis, 3 months after implantation of engineered cartilage, that there were no signs of inflammation; however, there was generally a failure of the implants to integrate laterally with the surrounding host cartilage (Fig. 5D, upper panels). Following the preliminary studies, cartilage engineered using iROR2+ve, ROR2–ve, or unfractionated MSCs from eight human donors was implanted into defect sites in this sheep model. The extent and quality of cartilage repair after 3 months were measured as percentage defect filling measured macroscopically by morphometric analysis and biochemical quality measured as the percentage GAG and percentage type II collagen in the repair tissue. Defect filling over 90% of the defect sites was observed in those animals treated using cartilage from iROR2+ve MSCs, whereas in those treated using cartilage from unfractionated or ROR2–ve MSCs, there was significantly less defect filling (Fig. 5E). Regenerated tissue was dissected from the implant site and analyzed for content of type II collagen and proteoglycans, in comparison with samples of the preimplantation tissue‐engineered cartilage, allowing an assessment of maturation of the implants over 3 months. There was a large and significant increase in the type II collagen content of implants, irrespective of which MSC fraction as used whereas the proteoglycans content decreased significantly during the same period in vivo. (Fig. 5D, lower panels). Overall, these data show improved quality of cartilage measured biochemically in all three treatment groups with no apparent advantage of iROR2+ve MSCs. However, there was clearly a greater abundance of repair tissue, with no loss of tissue quality, when using iROR2+ve MSCs compared with control groups, measured as defect filling, indicating the potential therapeutic advantage of this cell population.

### Native Origin of ROR2+ Cells

**Figure 6:**
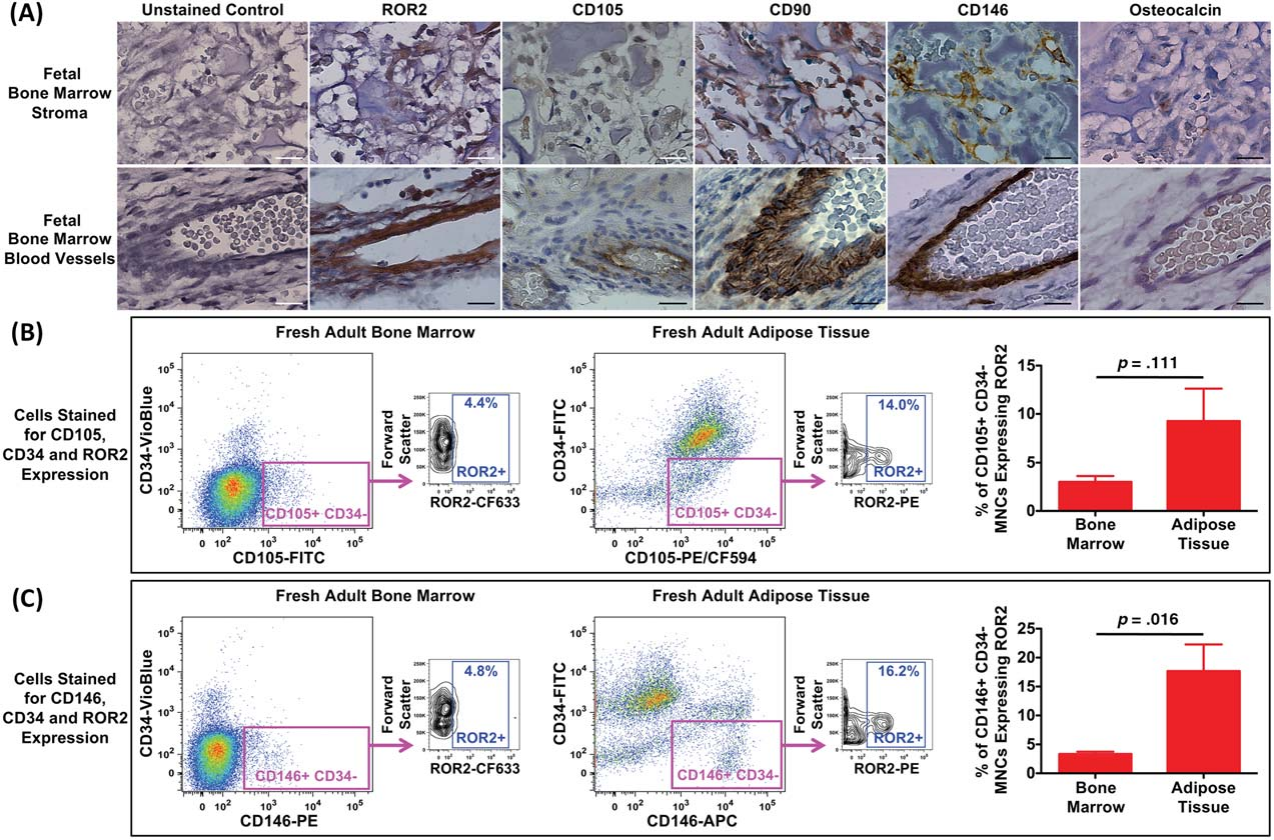
The in vivo and ex vivo origin of receptor tyrosine kinase‐like orphan receptor 2 (ROR2)+ve cells in human tissues. **(A):** Immunolocalisation of ROR2, in comparison with the mesenchymal stem cell marker proteins CD105 and CD90, the pericyte marker CD146 and the osteogenic marker osteocalcin, in the developing ulna limb bud from a human fetus of 11–12 weeks gestation. Regions of bone marrow stroma and blood vessels are shown. In all cases, DAB chromagen (brown) was used for visualization of positive signal and hematoxylin (purple) was used as a nuclear counter‐stain (scale bar = 20 μm). ROR2 expression was also analyzed in samples of fresh, uncultured adult human bone marrow and adipose tissue [panels **(B)** and **(C)**] to determine the proportion of cells expressing ROR2 on initial isolation. Mononuclear cells were isolated from fresh bone marrow (*n* = 5) or adipose tissue (*n* = 4) and immediately analyzed by flow cytometry following incubation with antibodies against CD105, CD146, CD34, and ROR2. Representative flow cytometry plots are shown and the graphs show the percentage of viable CD105+/CD34– [panel (B)] and CD146+/CD34– [panel (C)] cells that express ROR2. Comparison of differences between groups was by two‐tailed Mann‐Whitney *U* test. Abbreviations: APC, allophycocyanin; FITC, fluorescein isothiocyanate; PE, phycoerythrin; ROR2, receptor tyrosine kinase‐like orphan receptor 2.

We considered it important to determine whether the iROR2+ve cells observed in expanded, adherent MSCs are a consequence of tissue culture conditions or whether they exist in situ and in freshly isolated cells before adhesion to plastic for culture. Since ROR2 is thought to be important in limb bud development 23, sections of human fetal developing ulna limb bud (developing arm bones) were immunostained for ROR2 and for MSC‐related markers, CD105 and CD90. Since the natural precursor of MSCs in vivo is a perivascular cell, including the pericyte, the limb buds were also stained for the pericyte marker CD146. ROR2+ve cells were found in the bone marrow stroma as well as surrounding the blood vessels of limb bud from 11–12 week old human fetuses (Fig. 6A). These cells were colocated with cells positive for CD105 and CD90 as well as CD146, whereas there were no osteocalcin positive (bone‐forming) cells in these regions (Fig. 6A). These data support the theory that endogenous MSCs and their natural precursor, the pericyte, express ROR2 in development. Furthermore, a small number of ROR2+ve cells were also observed surrounding the blood vessels of adult human bone marrow tissue sections, again colocated with CD146+ve cells (Supporting Information Fig. S5), suggesting that ROR2+ve mesenchymal lineage cells are naturally occurring rather than resulting from tissue culture.

To further investigate the naturally occurring ROR2+ve mesenchymal cells in human adult tissues, we used flow cytometry to determine their occurrence in uncultured mononuclear cell populations that had been freshly isolated from adult bone marrow and adipose. Within the CD105+ve, CD34–ve MSC population (Fig. 6B), we observed a small percentage of ROR2+ve cells that was not significantly different between bone marrow (mean ± SEM of 3.0%±0.6%) and adipose (9.3% ± 3.4%). Within the CD146+ve, CD34–ve pericyte population (Supporting Information Fig. S6C), we also observed a small percentage of ROR2+ve cells in bone marrow (mean ± SEM of 3.3% ± 0.4%), but a significantly higher percentage in adipose (17.7% ± 4.6%). These data confirm that ROR2+ve MSCs/pericytes are naturally occurring. Furthermore, in adults, they are not restricted to bone marrow but can be found in at least one other site in the body.

Having established that ROR2 is expressed on low numbers of freshly isolated MSCs, we then determined how many passages were required to reach maximal ROR2 upregulation in culture. Bone marrow derived MSCs were seeded onto tissue culture plastic and analyzed for ROR2, after growing to high density, at the end of each passage. After initial adherence to and growth on tissue culture plastic there was a marked upregulation of iROR2, reaching a maximum at the end of passage 1 (Supporting Information Fig. S7). This coincides with a high capacity for cartilage formation at early passage (Supporting Information Fig. S3). Subsequently, there was a decrease in iROR2 expression at each passage that correlates with the gradual fall in chondrogenic capacity over multiple passages.

### ROR2 Changes with Age and Osteoarthritis

**Figure 7:**
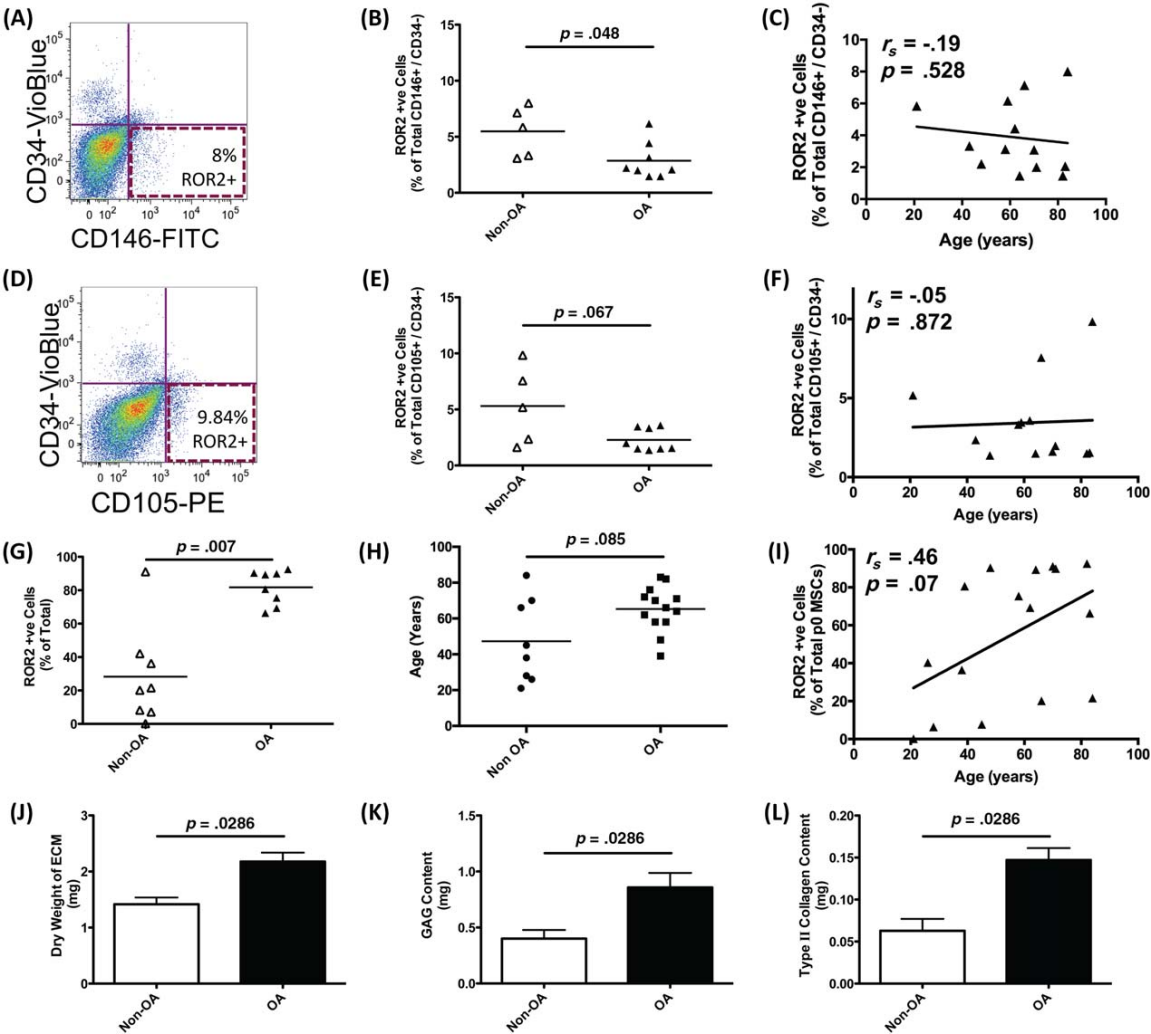
Variation in receptor tyrosine kinase‐like orphan receptor 2 (ROR2) with age and in patients with osteoarthritis (OA). In panels **(A–F)**, ROR2 expression was analyzed in samples of fresh, uncultured adult human bone marrow to determine the proportion of cells expressing ROR2. Mononuclear cells were isolated using Ficoll separation and immediately analyzed by flow cytometry following incubation with antibodies against CD105, CD34, and ROR2 or CD146, CD34, and ROR2. Representative flow cytometry plots are shown for both CD146+/CD34– pericytes (A) and CD105+/CD34– mesenchymal stem cells (MSCs) (D). The percentage of cells in fresh bone marrow which express ROR2 isolated from donors with and without OA (non‐OA *n* = 5, OA *n* = 8) are shown for both CD146+/CD34– pericytes (B) and CD105+/CD34– MSCs (E). Statistical analysis by Mann‐Whitney *U* test. The correlation of ROR2 expression with age of donor is shown for both CD146+/CD34– pericytes (C) and CD105+/CD34– MSCs (F). Spearman rank correlation coefficients are shown. In panels **(G–I)** ROR2 expression was analyzed by flow cytometry after isolation of MSCs and their subsequent expansion in vitro. Results are shown as the percentage of ROR2+ve cells at the time of first passage [p0, panel (G), statistical analysis by Mann Whitney *U* test] and as correlation with donor age [panel (I), Spearman rank correlation coefficient is shown]. The age‐range of patients in the non‐OA and OA groups is shown in panel h (statistical analysis by two‐tailed Mann‐Whitney *U* test). In panels **(J–L)**, results are shown for cartilage tissue engineering using MSCs isolated from either non‐OA (*n* = 4) or OA (*n* = 4) patients. Statistical analysis by Mann‐Whitney *U* test. Abbreviations: ECM, extracellular matrix; FITC, fluorescein isothiocyanate; MSC, mesenchymal stem cell; OA, osteoarthritis; PE, phycoerythrin; ROR2, receptor tyrosine kinase‐like orphan receptor 2.

Osteoarthritis (OA) is a disease of ageing in which there is typically a loss of articular cartilage as the disease progresses. We were, therefore, interested in whether there is a deficiency of ROR2 expression by MSCs in OA and whether this is related to increasing age. We first analyzed the ROR2 expression on freshly isolated CD146+ve, CD34–ve pericytes (Fig. 7A–7C) and CD105+ve, CD34–ve MSCs (Fig. 7D, 7E). MSCs/pericytes from OA patient bone marrow expressed significantly less ROR2 than MSCs/pericytes from non‐OA bone marrow (Fig. 7B, 7C) and this was not a feature of aging since there was no apparent correlation between age of the donor and ROR2 expression (Figs. 7E, 7F). In parallel experiments, we isolated plastic‐adherent MSCs from OA and non‐OA patients and stimulated maximal ROR2 expression by growing them to confluence before determining ROR2 by flow cytometry. There was a tendency for much higher expression of ROR2 in confluent OA MSCs compared with non‐OA (Fig. 7G), although this did not reach statistical significance. This might be explained in part by age of the patient since the age distribution for the OA donors was higher than the control group and there was a weak correlation between donor age and maximal ROR2 expression, although these differences were not significant (Figs. 7H, 7I). If OA MSCs do in fact express higher levels of ROR2 than non‐OA MSCs then they should also be better at generating cartilage. To test this hypothesis, we used MSCs from each group in cartilage tissue engineering and found that the OA MSCs produced cartilage with higher weight and greater proteoglycans and type II collagen content than non‐OA MSCs (Fig. 7J–7L).

## Discussion

Predictive functional MSC markers are critical for ensuring improved therapeutic outcomes and meeting regulatory demands when implanting MSC‐derived tissue engineering products into patients. It is widely recognized that the available MSC markers are not predictive of functional capacity and that new markers relating to subsets of this heterogeneous stem cell population will be required to move the field forward 24, 25. We have successfully used a genomic profiling strategy to identify an MSC cell surface marker that is predictive of enhanced chondrogenesis. Previous studies have focused on changes in gene expression after the initiation of specific differentiation pathways, or comparing gene expression patterns for MSCs isolated from different tissues 26. In contrast, we have studied the gene expression pattern of undifferentiated MSC clones defined according to their subsequent capacity to undergo chondrogenesis, so testing the predictive value of any genes that are differentially upregulated in those MSCs that are destined to be highly chondrogenic. This general approach could be applied to other cell differentiation pathways. While previous studies have provided some evidence for predictive chondrogenic markers in high‐density pellet cultures assays 27, we have been able to use iROR2 selection to generate an improved three‐dimensional tissue engineered cartilage that had greater efficacy in a sheep cartilage‐repair model than unfractionated MSCs and so could potentially be of therapeutic benefit in human patients.

The success of our cloning approach depended on our well defined cartilage tissue engineering protocol 11 and type II collagen immunoassay. We developed the assay originally as a method of studying osteoarthritic cartilage 28 and then further refined it for the study of cartilage regeneration quality in patient biopsies 29. Our quantitative analyses showed a wide range of cartilage tissue engineering outcomes from one clone to another with no apparent correlation to osteogenic or adipogenic potential (Supporting Information Table S4). Two previous studies have described the cloning of MSCs from bone marrow by limiting dilution 16, 30, and these are in agreement with our data (Supporting Information Fig. S1), indicating that more rapidly proliferating MSC clones are better able to differentiate. A total of 82 genes were found to be significantly upregulated in the highly chondrogenic MSCs compared with the poorly chondrogenic clones. Our gene and subsequent flow cytometry screening identified ROR2 as being upregulated on the surface of highly chondrogenic clones.

We were encouraged to investigate this molecule because of its well‐described importance in limb development 23, 31, 32, 33. ROR1 and ROR2 were first identified by Masiakowski and Carroll, who recognized the importance of tyrosine kinase in growth factor receptor function and, therefore, screened for novel genes encoding proteins with a tyrosine‐kinase like cytoplasmic domain 34. A series of important studies then identified ROR2 as playing a key role in cartilage and growth plate development in the limb buds of mice. DeChiara et al. 23 showed that ROR2 is selectively expressed in chondrocytes of the growth plate of long‐bones but not in those bones formed by intra‐membranous ossification, indicating a close relationship between growth plate cartilage and ROR2. They showed that mice homozygous for the mutant allele have disrupted growth of those bones that grow through endochondral ossification and they concluded that ROR2 is essential both for initial patterning of the cartilage anlagen and for subsequent regulation of mature cartilage 23. These observations have been replicated by other groups 32, 33, 35. ROR2 mutations have been described in patients with recessive Robinow syndrome 31, 36, which leads to limb shortening, abnormal development of the spinal vertebrae and brachydactyly (shortened digits), as well as craniofacial abnormalities. Therefore, ROR2 is a biologically relevant cell surface marker for cartilage formation as well as being of practical use in tissue engineering.

We were further encouraged to investigate this molecule because of its role as a receptor for Wnt5a 37, 38, 39, 40, 41, 42. This receptor requires tyrosine kinase activity to mediate noncanonical signaling 36, 38. However, the downstream signaling pathways are complex and only partially understood. Canonical Wnt signaling and activation of β‐catenin inhibits chondrogenesis, through binding of Sox‐9 to β‐catenin, so inhibiting upregulation of type II collagen synthesis 43. It has been demonstrated that Wnt‐5a signaling can antagonize the canonical Wnt signaling pathway by promoting β‐catenin degradation 44. This suggests that Wnt5a signaling through ROR2 has the potential to upregulate chondrogenesis through activation of SOX‐9. On the other hand, Wnt1 and Wnt3a can also bind to ROR2 and in this way activate the canonical pathway 37, 45, which might be expected to inhibit chondrogenesis. ROR2 has also been shown to play a key role in bone formation, as its expression is highly regulated during both osteoblast differentiation 46, 47 and osteoclastogenesis 48. Taken together, these studies indicate that ROR2 plays an important role in musculoskeletal development, but the mechanisms that are critical in vivo remain to be established.

Evidence that ROR2 expression is a feature of endogenous cells is provided by our immunohistochemical data describing ROR2+ve cells in the developing limb bud as well as in adult bone marrow, combined with our flow cytometry data demonstrating expression of ROR2 by pericytes in adult human bone marrow and adipose tissue. We have previously identified the pericyte as a precursor of MSCs found in bone marrow and other organs 1, 2. Our data, therefore, support a possible role for ROR2 in the chondrogenic function of pericytes as well as MSCs and suggest that this receptor has biological relevance.

The use of allogeneic MSCs as therapeutic tools in cartilage repair is limited by the variability of their chondrogenic capacity from one donor to the next and the loss of chondrogenic capacity with increasing passage (Supporting Information Fig. S3). Selection of iROR2+ve cells ensures a higher chondrogenic capacity and opens up the possibility of expanding MSCs through multiple PDs while maintaining an increased iROR2 expression and therefore enhanced chondrogenesis. The expanded cells could then be used to treat cartilage damage either by direct implantation into the lesions or indirectly, through injection into the synovial space.

## Conclusion

To conclude, iROR2 is associated with pericytes in situ and with MSCs ex vivo as well as with enhanced chondrogenesis and thus can be exploited as a biological marker. It could become central to the effective translation of MSC biology into therapeutic strategies for cartilage repair in patients with OA and other diseases of the joint and for extensive cartilage loss in the nose and ear following trauma or cancer.

## Author Contributions

S.C.D., K.B., A.S., and T.K.: collection and assembly of data, data analysis and interpretation, manuscript writing; C.A.S., R.L.W, L.W., and S.P.: collection and assembly of data; C.C.W. and D.E.: collection and assembly of data, data analysis and interpretation, manuscript writing, final approval of manuscript; R.F.d.G. and A.E.G.: undertook all experimental procedures in sheep including surgical implantation of tissue engineered cartilage, data analysis and interpretation, manuscript writing, final approval of manuscript; B.P.: conception and design, manuscript writing, final approval of manuscript; A.W.B.: provision of study material; W.K.: collection and/or assembly of data, manuscript writing, final approval of manuscript; A.P.H.: conception and design, financial support, manuscript writing, final approval of manuscript.

## Disclosure of Potential Conflicts of Interest

The authors indicated no potential conflicts of interest.

## Supporting information

Supporting Information Figure S1

Supporting Information Figure S2

Supporting Information Figure S3

Supporting Information Figure S4

Supporting Information Figure S5

Supporting Information Figure S6

Supporting Information Supplemental Methods

Supporting Information Table S1

Supporting Information Table S2

Supporting Information Table S3

Supporting Information Table S4

Supporting Information Table S5

Supporting Information Table S6

Supporting Information Table S7
