# Supplementary figures and images for: The Wnt5a Receptor, Receptor Tyrosine Kinase‐Like Orphan Receptor 2, Is a Predictive Cell Surface Marker of Human Mesenchymal Stem Cells with an Enhanced Capacity for Chondrogenic Differentiation
Source: Stem Cells. 2017 Aug 30;35(11):2280–91. doi: 10.1002/stem.2691 (PMC5707440; doi:10.1002/stem.2691)

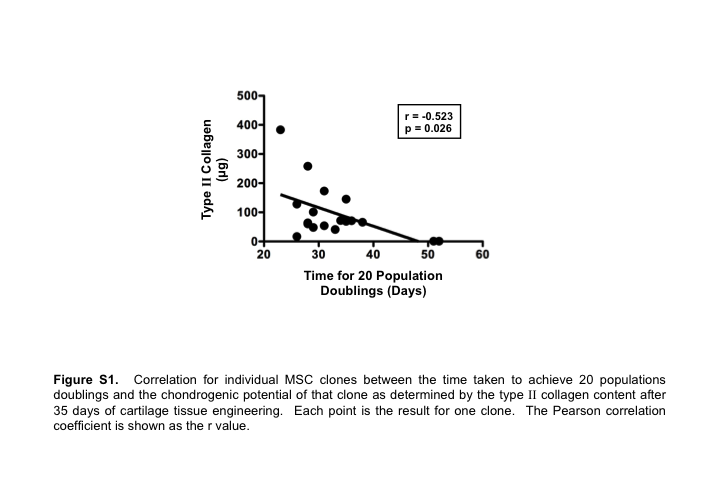

Supplement: Supplementary file 1 — Supporting Information Figure S1 [file STEM-35-2280-s001.tiff]

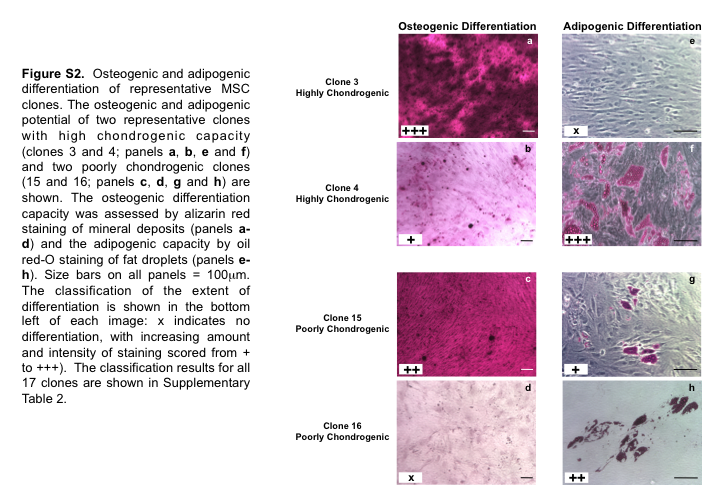

Supplement: Supplementary file 2 — Supporting Information Figure S2 [file STEM-35-2280-s002.tiff]

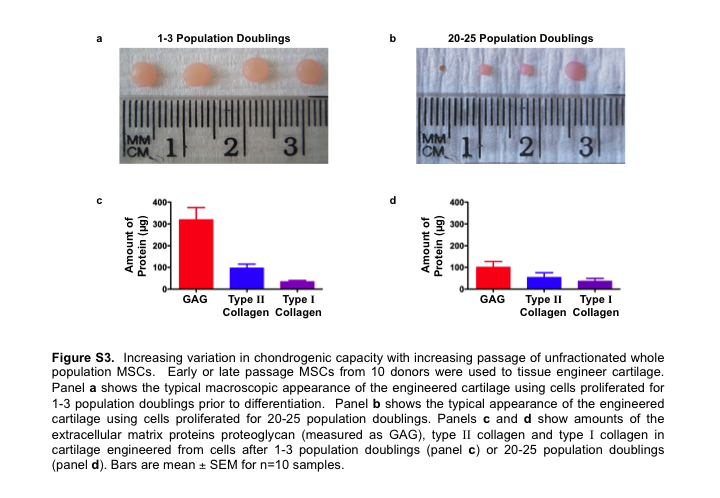

Supplement: Supplementary file 3 — Supporting Information Figure S3 [file STEM-35-2280-s003.tiff]

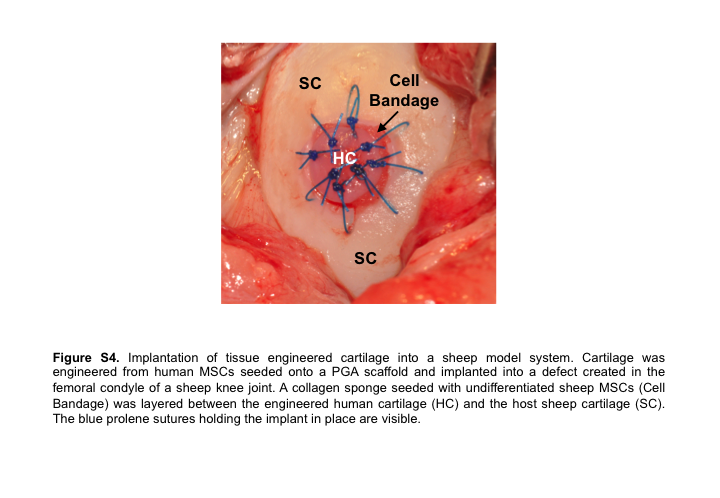

Supplement: Supplementary file 4 — Supporting Information Figure S4 [file STEM-35-2280-s004.tiff]

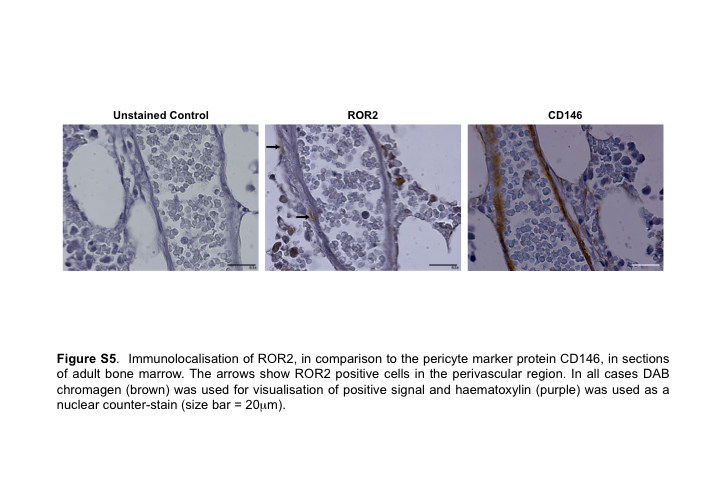

Supplement: Supplementary file 5 — Supporting Information Figure S5 [file STEM-35-2280-s005.tiff]

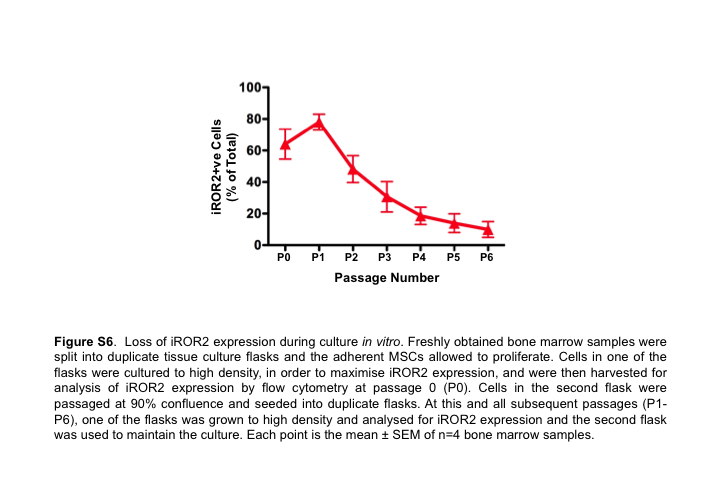

Supplement: Supplementary file 6 — Supporting Information Figure S6 [file STEM-35-2280-s006.tiff]
