# Supplementary material for: The Wnt5a Receptor, Receptor Tyrosine Kinase‐Like Orphan Receptor 2, Is a Predictive Cell Surface Marker of Human Mesenchymal Stem Cells with an Enhanced Capacity for Chondrogenic Differentiation
Source: Stem Cells. 2017 Aug 30;35(11):2280–91. doi: 10.1002/stem.2691 (PMC5707440; doi:10.1002/stem.2691)
Supplement: Supplementary file 7 — Supporting Information Supplemental Methods [file STEM-35-2280-s007.doc]

**The Wnt5a receptor ROR2 is a predictive cell surface marker of human mesenchymal stem cells with an enhanced capacity for chondrogenic differentiation**

**SUPPLEMENTAL METHODS**

**Flow chart of the phases of experimentation and different MSC preparations**


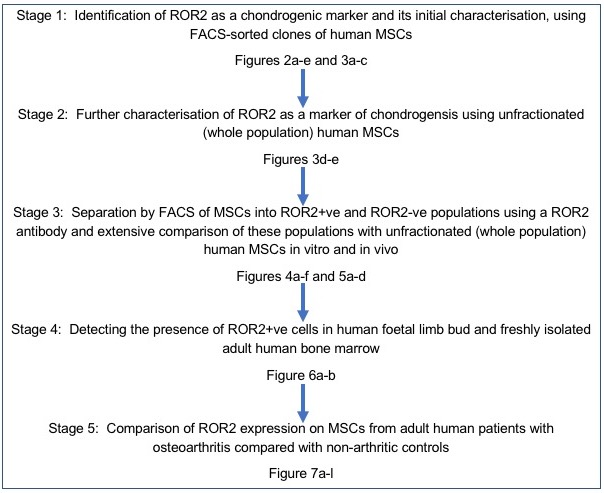


**Cloning**

Single-cell-derived clones were prepared from MSCs which had completed 1-3 PDs following thawing from liquid nitrogen stocks. A gate was set using forward scatter and side scatter parameters to include the majority of the cell population but to exclude cell debris and an Epics Altra flow cytometer (Beckman Coulter, High Wykombe, UK, [https://www.beckmancoulter.com](https://www.beckmancoulter.com/)) was used to deposit single MSCs into individual wells of 96-well tissue culture plates. The presence of single cells was confirmed using a microscope and any wells containing more than one cell were disregarded. Each clone was cultured in expansion medium supplemented with FGF-2. Clones which grew to 90% confluence were passaged and re-seeded in the same medium in 12-well plates. Expansion continued in 25, 75 and 175 cm^2^ flasks with the number of PDs and cellular senescence monitored at each passage.

**Chondrogenic differentiation**

*Cartilage tissue engineering*. The chondrogenic capacity of whole MSC populations or clonal lines was assessed by performing three-dimensional cartilage tissue engineering, as previously described . Briefly, 300,000 cells were loaded drop-wise onto 5 mm diameter x 2 mm thick polyglycolic acid (PGA) scaffold discs (Confluent Medical, Freemont, CA, USA, [https://confluentmedical.com](https://confluentmedical.com/)) which had been pre-coated with 100 μg/ml fibronectin (Sigma). Constructs were then cultured in chondrogenic differentiation medium consisting of DMEM, containing 4500 mg/L glucose (Sigma), supplemented with 10 ng/ml transforming growth factor-β3 (TGF-β3; R&D Systems, Abingdon, UK, [www.rndsystems.com](http://www.rndsystems.com/)), 100 nM dexamethasone, 80 µM ascorbic acid 2-phosphate, 1 mM sodium pyruvate, 100 units/ml penicillin, 100 µg/ml streptomycin (all from Sigma), 1% insulin-transferrin-selenium-G (ITS) and 2 mM Glutamax-I (both from Invitrogen). After 7 days, the medium was further supplemented with 10µg/ml bovine pancreatic insulin (Sigma) until the end of culture. The constructs were incubated at 37°C for a total of 35 days on a rotating platform and medium was changed every three days.

*Biochemical analysis*. Cartilage constructs were freeze-dried and weighed at the end of the 35 day tissue engineering period. The extracellular matrix was fully solubilised by overnight digestion with 2 mg/ml bovine pancreatic trypsin (Sigma) which was then boiled for 15 min to inhibit the action of the enzyme . In order to obtain the dry weight of extracellular matrix in the construct, remaining undigested scaffold material was freeze-dried, weighed and subtracted from the original dry weight. The amounts of type II collagen and proteoglycan in each digested cartilage construct were measured using specific assays. Type II collagen was quantified by inhibition ELISA using a mouse IgG monoclonal antibody to denatured type II collagen . Proteoglycan in the digests was measured as sulphated glycosaminoglycan (GAG) using a dimethylmethylene blue (Sigma) colourimetric assay .

**Osteogenic and adipogenic differentiation**

Whole MSC populations or MSC clones were grown in monolayer until 50-70% confluent prior to osteogenic differentiation or 100% confluent prior to adipogenic differentiation. In both cases, control cells were then cultured in α-MEM (Invitrogen) basal medium containing 10% FBS, 100 units/ml penicillin, 100 µg/ml streptomycin (all from Sigma) and 2 mM Glutamax-I (Invitrogen). Cells stimulated to undergo differentiation were cultured in basal medium containing either Osteogenic Supplement or Adipogenic Supplement (R&D Systems) for 21 days. Following osteogenic differentiation cells were fixed in 70% ethanol and stained with 40 mM alizarin red S (Sigma), pH4.1, for 5 min. Following adipogenic differentiation cells were fixed in 4% paraformaldehyde and stained with 0.3% oil red O (Sigma) for 30 min. The extent of differentiation was scored under blind conditions and classified as +, ++ or +++ according to increasing area and number of mineralised deposits following osteogenic differentiation and increasing number of lipid droplets following adipogenic differentiation.

**Gene array analysis**

The gene expression profiles of the four most chondrogenic and four least chondrogenic MSC clones were determined using microarray analysis. Total RNA was extracted from the undifferentiated clones using an RNeasy Micro Kit (Qiagen, Manchester, UK, [https://www.qiagen.com](https://www.qiagen.com/)) and the quantity and quality of the isolated RNA determined using an Agilent 2100 Bioanalyser (Agilent Technologies, Stockport, UK, [http://www.agilent.com](http://www.agilent.com/)). GeneChip Whole Transcript Sense Target Labelling Assay was used to generate amplified and biotinylated sense-strand DNA targets from the whole expressed genome. Fragmented and labelled DNA target was hybridised to a GeneChip Human Gene 1.0 ST array (Affymetrix, High Wycombe, UK, [http://www.affymetrix.com](http://www.affymetrix.com/)). Arrays were washed and stained using an Affymetrix Fluidics Station 450 and scanned using a GeneChip Scanner 3000. Expression Console software (Affymetrix) was used to generate probe set summarization files using the gene level default RMA-sketch workflow. Gene expression in the highly and poorly chondrogenic clones was compared using two-way ANOVA and only genes showing significant differences in expression were included in further analysis (p<0.05). Heat maps and dendograms were used to examine hierarchical clustering of genes which were then listed in decreasing order of statistical strength of differential expression to determine the genes which showed the greatest differences between the groups. Gene ontology was used to examine the functions of genes showing significant upregulation on highly chondrogenic clones and genes involved in signalling pathways were determined using the online Pathway Interaction Database (<http://pid.nci.nih.gov/>). Genes encoding proteins with membrane-spanning domains were also identified.

**Detection of cell surface proteins on MSC clones**

A single highly chondrogenic clone and a single poorly chondrogenic clone were tested by flow cytometry for the expression of several cell surface proteins identified following gene array analysis. The cells were incubated with antibodies against the cell-surface proteins STMN2, ASPN, PLXDC2, FGFR2, DCLK1 and ROR2. Any unconjugated primary antibodies were detected using fluorescein isothiocyanate (FITC) or phycoerythrin (PE) conjugated second antibodies (see **Supplemental Table S1** for antibody details). Cells were analysed using an Epics Altra flow cytometer (Beckman Coulter) after the addition of 2.5 µg/ml propidium iodide (PI) to exclude dead cells (Sigma). The percentage of cells expressing each protein was determined by comparison to background staining with appropriate isotype controls using FlowJo flow cytometry analysis software.

**Detection of ROR2 gene and protein expression**

Whole MSC populations or MSC clones were grown in monolayer on tissue culture plastic. RNA was extracted from some cultures using a Total RNA Purification Kit (Norgen Biotek, Thorold, ON, Canada, [https://norgenbiotek.com](https://norgenbiotek.com/)) and cDNA was synthesised using a PrimeScript RT Reagent Kit (TaKaRa, Mountain View, CA, USA, [http://www.clontech.com](http://www.clontech.com/)). The expression of the ROR2 gene was determined using specific primers (**Supplemental Table S2**) and SYBR Green I detection on a Rotor-Gene 6000 real-time PCR (Qiagen). Data was normalised to β-actin expression and analysed using REST 2009 gene quantification software (Qiagen). Cells were harvested from replicate cultures and incubated with 20 µg/ml anti-human ROR2 primary antibody. ROR2 protein on the cell surface was then detected using PE-conjugated goat anti-mouse IgG second antibody (**Supplemental Table S1**) and subsequent flow cytometry analysis.

**Effect of cell density on ROR2 protein expression**

MSCs were seeded at a low density of 2000 cells / cm^2^ in multiple tissue culture flasks and allowed to proliferate in expansion medium supplemented with FGF-2. Cells were grown to low density (3-6 days), confluence (7-14 days) or high density (14-18 days) before harvesting for analysis of ROR2 protein expression by flow cytometry. After 10 days of culture, when the cells had reached 100% confluence, some MSCs were re-plated at low-density (2700 cells / cm^2^) in expansion medium supplemented with FGF-2. After 1, 2 or 3 days of further culture, the cells were harvested and analysed for ROR2 expression by flow cytometry.

**Isolation of iROR2+ve and ROR2-ve MSCs by sterile cell sorting**

Undifferentiated whole population MSCs from 8 different donors were seeded at a high density of 20,000 cells / cm^2^ and cultured at confluence, in expansion medium containing FGF-2, for 7 days to allow maximum expression of ROR2 protein. Approximately 10x10^6^ cells from each donor were labelled with anti-human ROR2 primary antibody and PE-conjugated second antibody as described earlier. MSCs expressing iROR2 and those cells with no ROR2 protein upregulation in confluent culture were selected and collected using an Influx high-speed cell sorter (BD Biosciences, Oxford, UK, <http://www.bdbiosciences.com/eu/home>) following exclusion of non-viable cells staining positive with PI. Immediately after cell sorting, a small number of the separated cells were re-analysed by flow cytometry to determine the purity of the iROR2+ve and ROR2-ve populations. The remaining sorted cells, together with the whole unsorted MSC population from each donor, were cultured in expansion medium plus FGF-2 to obtain sufficient cells for further analysis.

**Characterisation of isolated iROR2+ve and ROR2-ve cells**

The three populations of cells from each donor (whole unsorted population, iROR2+ve and ROR2-ve cells) were tested for several properties as described below.

*Expression of known cell surface MSC markers.* The cells were incubated with antibodies against human CD105, CD90, CD73, VCAM1, STRO-1, CD146, CD271, CD34 and CD45 (see **Supplemental Table S1** for antibody details) and analysed by flow cytometry to determine the percentage of cells expressing each protein in comparison to background staining with appropriate isotype controls.

*Osteogenic and adipogenic differentiation.* Cells from each population were stimulated to undergo osteogenic or adipogenic differentiation as described earlier. Some cultures were stained with either alizarin red for matrix mineralisation or oil red to detect lipid droplets. RNA was extracted from replicate cultures and cDNA was synthesised as described earlier. The expression of the two osteogenic-specific genes integrin-binding sialoprotein (IBSP; bone sialoprotein) and alkaline phosphatase (ALPL) or the two adipogenic-specific genes lipoprotein lipase (LPL) and fatty acid binding protein 4 (FABP4) were determined by real-time PCR using specific primers (**Supplemental** **Table S2**). Data was normalised to β-actin expression.

*Growth kinetics and maintenance of phenotype*. Cells from each population were grown in continuous culture in expansion medium containing FGF-2. When cells reached 90% confluence they were passaged and re-seeded at low-density to allow continued cell proliferation. At each passage, the cells were counted and the cumulative number of PDs was plotted against time in culture to compare the growth kinetics of the different populations. In replicate cultures, cells were grown to high density at each passage after sorting to induce maximal expression of ROR2. At each time point, the percentage of cells expressing ROR2 was measured by flow cytometry as described earlier.

*In vitro chondrogenic capacity.* Isolated iROR2+ve and ROR2-ve cells, as well as unfractionated whole population, were seeded onto PGA scaffolds and cultured for 35 days as described earlier. The weights of the resulting tissue engineered cartilage constructs were measured and the amounts of the matrix components type II collagen and GAG were quantified using specific assays. Frozen sections of 7μm thickness were cut from replicate constructs and stained with haematoxylin and eosin (H&E) to assess overall extracellular matrix production and cellularity, or with 0.1% safranin O (Sigma) to evaluate GAG distribution (counterstained with 0.02% Fast Green F). Other sections were pre-treated with bovine testicular hyaluronidase (Sigma) and pronase (from *Streptomyces griseus*; Roche Applied Science, Burgess Hill, UK, [https://lifescience.roche.com](https://lifescience.roche.com/)) before being incubated with antibodies against type II collagen (see **Supplemental Table S1** for antibody details) as previously described . Antibodies were detected using biotinylated second antibodies and diaminobenzidine (DAB) substrate (Vector Laboratories, Peterborough, UK, [https://vectorlabs.com](https://vectorlabs.com/)). Normal goat IgG was used as a negative control and sections were counterstained with haematoxylin.

*In vitro T-cell immunoregulation assay.* Human peripheral blood mononuclear cells (PBMCs) were isolated from donor blood samples following centrifugation on 1.077 g/ml Ficoll-Paque (GE Healthcare Life Sciences, Little Chalfont, UK, http://www.gelifesciences.com) and cultured in RPMI-1640 containing L-glutamine and supplemented with 10% Human AB Serum and 100 units/ml penicillin, 100 μg/ml streptomycin (all from Sigma). PBMCs were stained with CellTrace™ Violet (ThermoFisher Scientific) to monitor T-cell proliferation. Labelled PBMCs were then stimulated with 3.75 μg/ml anti-human CD3 (HIT3a) and 2 μg/ml anti-human CD28 (CD28.2) (both from Affymetrix eBioscience, Cheshire, UK, [http://www.ebioscience.com](http://www.ebioscience.com/)) and co-cultured with isolated iROR2+ve, ROR2-ve and unfractionated MSCs for 72 hours. The T-cell proliferation profile for each population was analysed by flow cytometry following exclusion of non-viable cells stained with 7-amino-actinomycin D (7-AAD; BD Biosciences) and data was analysed using FlowJo analysis software.

**In vivo and ex vivo localisation of ROR2+ve cells**

*Immunostaining of bone marrow sections.* Paraffin-embedded sections of a developing ulna limb bud from a human foetus of 11-12 weeks gestation and bone marrow from a healthy adult donor (anonymous samples from the UCLA Translational Pathology Core Laboratory Tissue Bank) were incubated with antibodies against ROR2, CD105, CD90, CD146 and osteocalcin (see **Supplemental Table S1** for antibody details). Bound antibodies were detected using ImmPRESS anti-mouse or anti-rabbit Ig reagent and DAB peroxidase substrate (all from Vector Laboratories). All sections were counterstained with haematoxylin.

*Flow cytometry analysis of uncultured bone marrow and adipose tissue.* Bone marrow samples were obtained from 5 donors immediately following orthopaedic surgery. Mononuclear cells (MNCs) were isolated following centrifugation on 1.077 g/ml Ficoll-Paque (Stem Cell Technologies, Cambridge, UK, [https://www.stemcell.com](https://www.stemcell.com/)). Subcutaneous adipose tissue was harvested from 4 female donors during elective abdominoplasty or liposuction. All patients gave informed consent and the study was performed in full accordance with local ethics guidelines (South East Scotland Research Ethics Committee Ref 10/S1103/45). Adipose tissue was digested with 1mg/ml type II collagenase (Sigma) as previously described . MNCs from both bone marrow and adipose were stained with antibodies against CD105, CD146, CD34 and ROR2 (see **Supplemental Table S1** for antibody details) and analysed by flow cytometry following exclusion of non-viable cells stained with 7-amino-actinomycin D (7-AAD; BD Biosciences). Gates were set using fluorescence minus one (FMO) and isotype controls and data was analysed using FlowJo analysis software.

*Upregulation of ROR2 with culture.* Fresh bone marrow samples from 4 donors were added into duplicate tissue culture flasks containing expansion medium plus FGF-2. Adherent MSCs in one of the flasks were cultured to high density to maximise ROR2 expression before being harvested for analysis of ROR2 by flow cytometry. Cells in the second flask was passaged at 90% confluence and seeded into duplicate flasks. At this and all subsequent passages, one of the flasks was grown to high density and analysed for ROR2 expression and the second flask was used to maintain the culture.

**Sheep operative procedures**

Bone marrow was aspirated from the iliac crest , approximately 2 weeks prior to the cartilage surgery. Following sedation and general anaesthesia, the sheep were placed in right lateral recumbency. Wool was shaved around the left pos-posterior superior iliac crest and the skin was prepared with clorehexidine scrub. A small incision was made over the aspiration site, and a pre-heparinized 11-gauge Jamshidi needle (Rocket Medical, Watford, UK) inserted into the bone. A pre-heparinized syringe (0.5 ml, 1000 units/ml) was attached and approximately 5 ml bone marrow aspirated. The aspirate was gently agitated to mix with heparin inside the syringe before transfer to a universal tube and expeditious transport to the laboratory in a cool box.

A 6mm diameter chondral defect was prepared in the left medial femoral condyle of each of the sheep from which the bone marrow samples were obtained. The defect was created in the weight bearing area using a biopsy punch to create a sharp dissection and a small osteotome to remove soft tissue down to the calcified cartilage. We used a parapatellar approach to the medial condyle, without patellar luxation, with the animals in lateral recumbency and the operated limb in supine and maximally bent position.  The fat pad was not removed and the synovium and joint capsule were closed using one layer of continuous suture with vycril 3-0.

The defect was created such that its centre was located on the midline of the medial condyle, approximately 2cm caudal to the cranial border of the cartilage (which is the weight bearing area in sheep). In the area of the implant the sheep cartilage is around 1mm thick. We positioned the Cell Bandage first and the implant was then placed on top of it and the Cell Bandage was cut to fit the 6mm defect. The PGA constructs ranged from 4.5-6mm diameter. The collagen sponge used to create the cell bandage is soft and pliable once wet and so it was easily compressed and held in place underneath the sutured PGA construct. The Figure below summarises the positioning of cell Bandage and engineered cartilage.


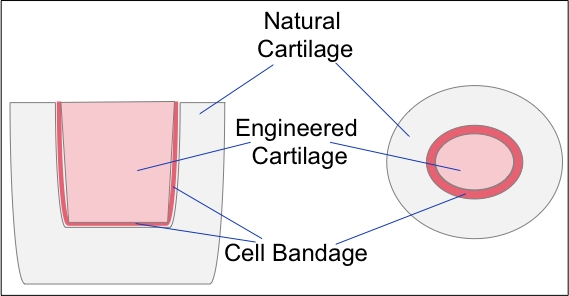


**Diagram showing positioning of Cell Bandage and engineered implants**

Engineered cartilage constructs produced from iROR2+ve cells, ROR2-ve cells or unfractionated whole population cells were randomly allocated to different sheep and the veterinary surgeon performing the implantations was blinded to the group allocation. Post-operatively the sheep were housed in small individual pens.

We used non-resorbable proline 6-0 sutures as we wanted to be sure that the implant would remain in contact with the surrounding cartilage for as long as possible to increase the chances of healing and avoid delamination. Despite this, there was no evidence of any adverse response to the implant. On clinical examination, at 1 week, 4 weeks and 12 weeks of post-op no lameness or joint swelling, localise heat or pain on palpation were identified. Furthermore, during post-mortem examination, no inflammation was seen on macroscopic examination of the tibial plateau, meniscus, patella, synovial fluid or synovium. The synovium was also examined microscopically and no inflammation signs were seen. No osteophytes, laxity, synovitis, effusion or any other signs of osteoarthritis were observed.

**Analysis of Cartilage formation in the sheep model**

The extent of defect filling was quantified by two independent observers using ImageJ analysis software (National Institutes of Health). The implanted human cartilage was dissected from the surrounding ovine tissue and the amounts of the matrix components type II collagen and GAG were quantified using specific assays as described above. Samples were analysed under blinded conditions and were randomised until the completion of data analysis.

**Accession Code**

Microarray data from this study have been deposited in the NCBI Gene Expression Omnibus database and are accessible through GEO Series accession number GSE54684.

**Statistical analysis**

Data were tested for Gaussian distribution using the Shapiro-Wilk test. Non-parametric analyses were used for those Figures where the number of samples was too low to test for normality. For those data sets where normality was proven, parametric analyses were used. Accordingly, comparison of differences was performed using the two-tailed Mann-Whitney *U* test, the Wilcoxon matched pairs test or the two-tailed paired t test. Comparison of more than two groups was by Kruskal-Wallis one-way analysis of variance with post-hoc bonferroni correction for multiple comparisons. When significant variance was demonstrated, differences between individual groups were then determined using the two-tailed Mann-Whitney U test. Correlation of ROR2 expression with other parameters was using the Spearman rank correlation coefficient (r_s_).

**References for Methods**
