# Supplementary material for: The Wnt5a Receptor, Receptor Tyrosine Kinase‐Like Orphan Receptor 2, Is a Predictive Cell Surface Marker of Human Mesenchymal Stem Cells with an Enhanced Capacity for Chondrogenic Differentiation
Source: Stem Cells. 2017 Aug 30;35(11):2280–91. doi: 10.1002/stem.2691 (PMC5707440; doi:10.1002/stem.2691)
Supplement: Supplementary file 8 — Supporting Information Table S1 [file STEM-35-2280-s008.doc]

**Table S1**

**Primary antibodies for flow cytometry**

| **Antigen** | **Antigen Symbol** | **Manufacturer** | **Catalogue Number** | **Isotype** | **Conjugated?** |
| --- | --- | --- | --- | --- | --- |
| Receptor tyrosine kinase-like orphan receptor 2 | ROR2 | R&D Systems | MAB2064 | Mouse IgG1 | No |
| Receptor tyrosine kinase-like orphan receptor 2 | ROR2 | R&D Systems | MAB2064 | Mouse IgG1 | CF633 * |
| Stathmin-like 2 | STMN2 | Sigma | WH0011075M2 | Mouse IgG2a | No |
| Asporin | ASPN | Abcam | ab58741 | Rabbit IgG | No |
| Plexin domain containing 2 | PLXDC2 | Abnova | H00084898-M01 | Mouse IgG2b | No |
| Fibroblast growth factor receptor 2 | FGFR2 | R&D Systems | FAB684F | Mouse IgG1 | FITC |
| Doublecortin-like kinase 1 | DCLK1 | Abcam | ab37994 | Rabbit IgG | No |
| Endoglin | CD105 | R&D Systems | FAB10971F | Mouse IgG1 | FITC |
| Endoglin | CD105 | BD Biosciences | 562380 | Mouse IgG1 | PE-CF594 |
| Thy-1 cell surface antigen | CD90 | R&D Systems | FAB2067P | Mouse IgG2a | PE |
| 5’-Nucleotidase | CD73 | R&D Systems | FAB5795P | Mouse IgG2b | PE |
| Vascular cell adhesion molecule 1 (CD106) | VCAM1 | R&D Systems | BBA22 | Mouse IgG2a | FITC |
| STRO-1 | STRO-1 | R&D Systems | MAB1038 | Mouse IgM | No |
| Melanoma cell adhesion molecule | CD146 | R&D Systems | FAB932P | Mouse IgG1 | PE |
| Melanoma cell adhesion molecule | CD146 | AbD Serotec | MCA2141A647 | Mouse IgG1 | APC |
| Nerve growth factor receptor | CD271 | R&D Systems | FAB367P | Mouse IgG1 | PE |
| CD34 | CD34 | eBioscience | 11-0349-42 | Mouse IgG1 | FITC |
| CD34 | CD34 | BD Biosciences | 555821 | Mouse IgG1 | FITC |
| CD34 | CD34 | Miltenyi Biotec | 130-095-393 | Mouse IgG2a | VioBlue |
| CD45 | CD45 | R&D Systems | FAB1430P | Mouse IgG1 | PE |

* Conjugate prepared using Mix-n-Stain CF633 Antibody Labelling Kit (Biotium; Cat No 92237)

**Supplementary Table 6 cont**

**Secondary antibodies for flow cytometry**

| **Second Antibody** | **Manufacturer** | **Catalogue Number** | **Conjugated?** |
| --- | --- | --- | --- |
| Goat anti-mouse IgG | R&D Systems | F0103B | FITC |
| Goat anti-mouse IgG | R&D Systems | F0102B | PE |
| Goat anti-rabbit IgG | R&D Systems | F0112 | FITC |
| Goat anti-mouse IgM | R&D Systems | F0118 | FITC |

Primary antibodies for immunohistochemical staining

| **Antigen** | **Manufacturer** | **Catalogue Number** | **Isotype** |
| --- | --- | --- | --- |
| Type II Collagen | Southern Biotech | 1320-01 | Goat IgG |
| ROR2 | Abgent | AP7672a | Rabbit IgG |
| CD105 | Abcam | ab114052 | Mouse IgG1 |
| CD90 | BD Biosciences | 555593 | Mouse IgG1 |
| CD146 | Abcam | ab75769 | Rabbit IgG |
| Osteocalcin | Santa Cruz | sc-30044 | Rabbit IgG |

**Secondary antibody kits for immunohistochemical staining**

VECTASTAIN Elite ABC Kit (Goat IgG) – Vector Laboratories PK-6105

ImmPRESS Anti-rabbit Ig Peroxidase Kit – Vector Laboratories MP-7401

ImmPRESS Anti-mouse Ig Peroxidase Kit – Vector Laboratories MP-7422-15
