# Supplementary material for: The Wnt5a Receptor, Receptor Tyrosine Kinase‐Like Orphan Receptor 2, Is a Predictive Cell Surface Marker of Human Mesenchymal Stem Cells with an Enhanced Capacity for Chondrogenic Differentiation
Source: Stem Cells. 2017 Aug 30;35(11):2280–91. doi: 10.1002/stem.2691 (PMC5707440; doi:10.1002/stem.2691)
Supplement: Supplementary file 9 — Supporting Information Table S2 [file STEM-35-2280-s009.doc]

**Table S2.** Primers used for quantitative real-time PCR

| **Gene Name** | **Gene Symbol** | **Forward Primer Sequence**  **(5’-3’)** | **Reverse Primer Sequence**  **(5’-3’)** | **Product Length**  **(base pairs)** |
| --- | --- | --- | --- | --- |
| Receptor tyrosine kinase-like orphan receptor 2 | ROR2 | AAACCCACCCCCTAA  CGTG | GCAGTCGTGAACCAT  ATTCTGT | 101 |
| Integrin-binding sialoprotein | IBSP | AAACGAAGAAAGCGA  AGCAGAA | GCTGCCGTTGCCGTT  TT | 94 |
| Alkaline phosphatase | ALPL | CGGAACTCCTGACCC  TTGAC | TGTTCAGCTCGTACT  GCATGTC | 84 |
| Lipoprotein lipase | LPL | ACAAGAGAGAACCAG  ACTCCAA | AGGGTAGTTAAACTC  CTCCTCC | 149 |
| Fatty acid binding protein 4 | FABP4 | AGCACCATAACCTTA  GATGGGG | CGTGGAAGTGACGCC  TTTCA | 132 |
| β-Actin | ACTB | GACAGGATGCAGAAG  GAGATTACT | TGATCCACATCTGCT  GGAAGGT | 142 |
