# Supplementary material for: The Wnt5a Receptor, Receptor Tyrosine Kinase‐Like Orphan Receptor 2, Is a Predictive Cell Surface Marker of Human Mesenchymal Stem Cells with an Enhanced Capacity for Chondrogenic Differentiation
Source: Stem Cells. 2017 Aug 30;35(11):2280–91. doi: 10.1002/stem.2691 (PMC5707440; doi:10.1002/stem.2691)
Supplement: Supplementary file 10 — Supporting Information Table S3 [file STEM-35-2280-s010.doc]

**Table S3**. Growth of MSC clones derived from the bone marrow of 6 patients. Initial cloning was by FACS sorting.

| **Patient Number** | **Number of clones added to 96-well plates** | **Number of clones expanded into**  **12-well plates** | **Number of clones expanded into**  **25cm^2^ flasks** | **Number of clones expanded into 75cm^2^ or 175cm^2^ flasks** | **Number of clones successfully undergoing 20 population doublings** |
| --- | --- | --- | --- | --- | --- |
| **PN1** | 96 | 4 | 3 | - | 0 |
| **PN2** | 96 | - | - | - | 0 |
| **PN3** | 96 | 17 | 8 | 3 | 3 |
| **PN4** | 96 | - | - | - | 0 |
| **PN5** | 240 | 40 | 38 | 34 | 21 |
| **PN6** | 240 | 22 | 19 | 11 | 1 |
