# Supplementary material for: The Wnt5a Receptor, Receptor Tyrosine Kinase‐Like Orphan Receptor 2, Is a Predictive Cell Surface Marker of Human Mesenchymal Stem Cells with an Enhanced Capacity for Chondrogenic Differentiation
Source: Stem Cells. 2017 Aug 30;35(11):2280–91. doi: 10.1002/stem.2691 (PMC5707440; doi:10.1002/stem.2691)
Supplement: Supplementary file 11 — Supporting Information Table S4 [file STEM-35-2280-s011.doc]

**Table S4.** Chondrogenic (amount of type II collagen in engineered cartilage), osteogenic (extent of alizarin red staining) and adipogenic (extent of oil red-O staining) differentiation capacity of MSC clones (x indicates no differentiation, with increasing amount and intensity of staining scored from + to +++; see Supplementary Figure 2 for examples).

| **Clone**  **Number** | **Amount of TII collagen in engineered cartilage (μg)** | **Extent of osteogenic differentiation** | **Extent of adipogenic differentiation** |
| --- | --- | --- | --- |
| **1** | 383 | **+++** | **+** |
| **2** | 258 | **+** | **+** |
| **3** | 173 | **+++** | x |
| **4** | 145 | **+** | **+++** |
| **5** | 128 | x | **++** |
| **6** | 101 | **+** | x |
| **7** | 72 | x | **+** |
| **8** | 71 | **++** | x |
| **9** | 69 | **++** | x |
| **10** | 66 | **++** | **+** |
| **11** | 64 | x | **+** |
| **12** | 60 | **+** | **+** |
| **13** | 54 | **+** | x |
| **14** | 48 | **++** | **+** |
| **15** | 17 | **++** | **+** |
| **16** | 1 | x | **++** |
| **17** | 1 | **+** | **++** |
