# Supplementary material for: The Wnt5a Receptor, Receptor Tyrosine Kinase‐Like Orphan Receptor 2, Is a Predictive Cell Surface Marker of Human Mesenchymal Stem Cells with an Enhanced Capacity for Chondrogenic Differentiation
Source: Stem Cells. 2017 Aug 30;35(11):2280–91. doi: 10.1002/stem.2691 (PMC5707440; doi:10.1002/stem.2691)
Supplement: Supplementary file 12 — Supporting Information Table S5 [file STEM-35-2280-s012.doc]

**Table S5.** Genes analysed in Figure 2a that are involved in signaling pathways (as determined using the online Pathway Interaction Database; http://pid.nci.nih.gov/).

| **Signaling Pathway** | **Genes** |
| --- | --- |
| Complement pathway | C7 C4A CFB C1R |
| Wnt signaling pathway | ROR2 JUN |
| FGF signaling pathway | FGFR2 JUN |
| Notch signaling | HEY2 JUN |
| IL-6 signaling | A2M JUN |
| Integrin signaling | THBS2 JUN |
| PDGF receptor signaling | PDGFD JUN |
| Syndecan-3-mediated signaling | PTN |
| Eicosanoid metabolism | PTGFR |
| p73 transcription factor network | HEY2 |
| Liver kinase B1 signaling | MAP2 |
| Arf6 trafficking | CPE |
| Prothrombin activation pathway | PROS1 |
