# Supplementary material for: The Wnt5a Receptor, Receptor Tyrosine Kinase‐Like Orphan Receptor 2, Is a Predictive Cell Surface Marker of Human Mesenchymal Stem Cells with an Enhanced Capacity for Chondrogenic Differentiation
Source: Stem Cells. 2017 Aug 30;35(11):2280–91. doi: 10.1002/stem.2691 (PMC5707440; doi:10.1002/stem.2691)
Supplement: Supplementary file 13 — Supporting Information Table S6 [file STEM-35-2280-s013.doc]

**Table S6. The 82 genes found to be significantly up-regulated by at least 1.3-fold on highly chondrogenic MSC clones in comparison to poorly chondrogenic clones (p<0.05; two-way ANOVA) as determined by whole genome microarray analysis. Genes are listed in decreasing order of statistical strength of differential expression (ANOVA).**

| **Gene**  **Name** | **Name in Figure 2** | **Fold Change** | **ANOVA**  **p-value** | **Gene Function** |
| --- | --- | --- | --- | --- |
| Zinc finger protein 204, pseudogene | ZNF204P | 1.35 | 0.0002 | Unknown |
| ST8 alpha-N-acetyl-neuraminide alpha-2,8-sialyltransferase 4 | ST8SIA4 | 2.14 | 0.0005 | Nervous system development |
| Osteomodulin | OMD | 1.61 | 0.0005 | Cell adhesion, biomineralisation |
| Rho GTPase activating protein 28 | ARHGAP28 | 1.95 | 0.0007 | Signal transduction |
| Guanylate binding protein 2, interferon-inducible | GBP2 | 1.30 | 0.0011 | Immune response |
| Phospholipid transfer protein | PLTP | 1.43 | 0.0016 | Lipid transport |
| Zinc finger protein 608 | ZNF608 | 1.41 | 0.0017 | Unknown |
| Pleiomorphic adenoma gene 1 | PLAG1 | 1.34 | 0.0017 | Transcription |
| Complement component 7 | C7 | 1.54 | 0.0022 | Complement activation |
| SET binding protein 1 | SETBP1 | 1.43 | 0.0027 | Unknown |
| Zinc finger protein 404 | ZNF404 | 1.35 | 0.0028 | Transcription |
| Leucine rich repeat containing 1 | LRRC1 | 1.32 | 0.0031 | Unknown |
| Small Cajal body-specific RNA 9 | SCARNA9 | 1.41 | 0.0031 | Unknown |
| Guanylate cyclase 1, soluble, beta 3 | GUCY1B3 | 1.34 | 0.0033 | cGMP biosynthesis, blood circulation |
| Platelet-derived growth factor receptor-like | PDGFRL | 1.41 | 0.0033 | Platelet activating factor receptor |
| Microtubule-associated protein 2 | MAP2 | 1.34 | 0.0046 | Negative regulation of microtubule depolymerisation |
| NHS-like 2 | NHSL2 | 1.34 | 0.0056 | Unknown |
| Alpha-2-macroglobulin | A2M | 2.02 | 0.0057 | Intracellular protein transport |
| Podocan-like 1 | PODNL1 | 1.40 | 0.0059 | Unknown |
| Similar to zinc finger protein 813 | LOC344787 | 1.31 | 0.0059 | Transcription |
| Dermatopontin | DPT | 1.40 | 0.0061 | Cell adhesion |
| Osteoglycin | OGN | 1.74 | 0.0061 | Bone formation |
| Tetraspanin 18 | TSPAN18 | 2.09 | 0.0061 | Transport |
| Sulphatase 2 | SULF2 | 1.72 | 0.0065 | Heparan sulphate proteoglycan metabolic process |
| Chromosome 21 open reading frame 91 | C21orf91 | 1.32 | 0.0078 | Unknown |
| G protein-coupled receptor 125 | GPR125 | 1.32 | 0.0080 | Neuropeptide signalling |
| Seizure related 6 homolog (mouse)-like 2 | SEZ6L2 | 1.45 | 0.0080 | Unknown |
| Plexin domain containing 2 | PLXDC2 | 1.74 | 0.0080 | Multicellular organismal development |
| Integral membrane protein 2C | ITM2C | 1.40 | 0.0081 | Unknown |
| AE binding protein 1 | AEBP1 | 1.33 | 0.0097 | Skeletal development, cell adhesion |
| NEDD4 binding protein 2 | N4BP2 | 1.30 | 0.0103 | Unknown |
| Peroxisome proliferator-activated receptor gamma, coactivator 1 alpha | PPARGC1A | 1.51 | 0.0108 | Temperature homeostasis, glucose homeostasis |
| SERTA domain containing 4 | SERTAD4 | 1.74 | 0.0113 | Unknown |
| Centrosomal protein 95kDa | CEP95 | 1.35 | 0.0115 | Unknown |
| Kelch-like 24 (Drosophila) | KLHL24 | 1.36 | 0.0116 | Unknown |
| miR-199a-2 stem-loop | miR-199a-2 | 1.32 | 0.0124 | Unknown |
| Leucine-rich repeats and immunoglobulin-like domains 3 | LRIG3 | 1.43 | 0.0128 | Unknown |

| Calcitonin receptor-like | CALCRL | 1.53 | 0.0142 | G-protein signalling coupled to cyclic nucleotide second messenger, smooth muscle cell proliferation |
| --- | --- | --- | --- | --- |
| Platelet derived growth factor D | PDGFD | 2.07 | 0.0152 | Cell proliferation |
| Complement component 1, r subcomponent | C1R | 1.33 | 0.0153 | Complement activation |
| Deleted in bladder cancer 1 | DBC1 | 1.52 | 0.0154 | Cell cycle arrest |
| Doublecortin-like kinase 1 | DCLK1 | 1.55 | 0.0163 | Central nervous system development |
| Fibroblast growth factor receptor 2 | FGFR2 | 1.61 | 0.0165 | Cell proliferation and differentiation |
| Basic helix-loop-helix family, member e41 | BHLHE41 | 1.40 | 0.0165 | Tumour suppressor gene |
| Leucine rich repeat containing 17 | LRRC17 | 1.52 | 0.0166 | Bone homeostasis |
| Stathmin-like 2 | STMN2 | 2.31 | 0.0167 | Neuron differentiation |
| Complement factor B | CFB | 1.33 | 0.0168 | Complement activation |
| Cystatin A (stefin A) | CSTA | 1.54 | 0.0170 | Keratinocyte differentiation |
| Phosphoglycerate dehydrogenase | PHGDH | 1.45 | 0.0173 | Amino acid biosynthesis |
| Gamma-glutamyltransferase 5 | GGT5 | 1.33 | 0.0188 | Amino acid metabolism |
| Collectin sub-family member 12 | COLEC12 | 1.67 | 0.0216 | Innate immune response, phagocytosis |
| Potassium channel, subfamily T, member 2 | KCNT2 | 1.44 | 0.0220 | Potassium ion transport |
| Microfibrillar-associated protein 4 | MFAP4 | 1.81 | 0.0221 | Cell adhesion |
| DNA-damage-inducible transcript 4-like | DDIT4L | 1.79 | 0.0221 | Negative regulation of signal transduction |
| Matrilin 2 | MATN2 | 1.36 | 0.0228 | Unknown |
| Contactin associated protein-like 2 | CNTNAP2 | 1.52 | 0.0244 | Cell adhesion, transmission of nerve impulse |
| Asporin | ASPN | 1.85 | 0.0254 | Cartilage homeostasis |
| Protein S (alpha) | PROS1 | 1.31 | 0.0256 | Blood coagulation |
| Carboxypeptidase E | CPE | 1.32 | 0.0265 | Proteolysis, neuropeptide signalling, insulin processing |
| Jun proto-oncogene | JUN | 1.33 | 0.0270 | Transcription, differentiation |
| Prostaglandin F receptor (FP) | PTGFR | 1.70 | 0.0274 | G-protein coupled receptor protein signalling pathway |
| Similar to CG3558-PA, isoform A | LOC729830 | 1.33 | 0.0300 | Unknown |
| CKLF-like MARVEL transmembrane domain containing 8 | CMTM8 | 1.43 | 0.0303 | Chemotaxis |
| Interferon induced transmembrane protein 1 | IFITM1 | 1.49 | 0.0316 | Negative regulation of cell proliferation |
| Odd-skipped related 2 (Drosophila) | OSR2 | 1.70 | 0.0328 | Cell proliferation, embryonic skeletal morphogenesis, palate development |
| Slit homolog 3 (Drosophila) | SLIT3 | 1.30 | 0.0330 | Multicellular organismal development, nervous system development, differentiation |
| Cysteine dioxygenase, type I | CDO1 | 1.45 | 0.0342 | Amino acid biosynthesis, inflammatory response |
| Receptor tyrosine kinase-like orphan receptor 2 | ROR2 | 1.30 | 0.0347 | Skeletal development, cartilage condensation, Wnt receptor signalling, multicellular organismal development, differentiation |
| Thrombospondin 2 | THBS2 | 1.32 | 0.0356 | Cell adhesion |
| Long intergenic non-protein coding RNA 478 | LINC00478 | 1.34 | 0.0359 | Unknown |
| Lipopolysaccharide-induced TNF factor | LITAF | 1.50 | 0.0406 | Transcription, apoptosis, TNFα expression |

| Glucoside xylosyltransferase 2 | GXYLT2 | 1.38 | 0.0415 | Glucosyl transferase |
| --- | --- | --- | --- | --- |
| Serpin peptidase inhibitor, clade F (alpha-2 antiplasmin, pigment epithelium derived factor), member 1 | SERPINF1 | 1.46 | 0.0422 | Multicellular organismal development, cell proliferation, neurogenesis |
| Pleiotrophin (heparin binding growth factor 8, neurite growth-promoting factor 1) | PTN | 1.71 | 0.0424 | Nervous system development, cell proliferation, bone mineralisation |
| Extracellular matrix protein 2, female organ and adipocytes specific | ECM2 | 1.45 | 0.0430 | Cell matrix adhesion |
| Sema domain, immunoglobulin domain (Ig), short basic domain, secreted, (semaphorin) 3A | SEMA3A | 1.44 | 0.0435 | Multicellular organismal development, cell differentiation, neurogenesis |
| Tet methylcytosine dioxygenase 1 | TET1 | 1.34 | 0.0436 | Unknown |
| Hairy/enhancer-of-split related with YRPW motif 2 | HEY2 | 1.41 | 0.0460 | Notch signalling pathway, multicellular organismal development, nervous system development |
| Phospholipase C-like 1 | PLCL1 | 1.42 | 0.0485 | Lipid metabolism |
| Complement component 4A (Rodgers blood group) | C4A | 1.41 | 0.0487 | Inflammatory response, complement activation |
| Small nucleolar RNA, C/D box 116-6 | SNORD116-6 | 1.37 | 0.0487 | RNA splicing |
| Zinc finger protein 560 | ZNF560 | 1.36 | 0.0490 | Transcription |
