# Supplementary material for: The Wnt5a Receptor, Receptor Tyrosine Kinase‐Like Orphan Receptor 2, Is a Predictive Cell Surface Marker of Human Mesenchymal Stem Cells with an Enhanced Capacity for Chondrogenic Differentiation
Source: Stem Cells. 2017 Aug 30;35(11):2280–91. doi: 10.1002/stem.2691 (PMC5707440; doi:10.1002/stem.2691)
Supplement: Supplementary file 14 — Supporting Information Table S7 [file STEM-35-2280-s014.doc]

**Table S7.** Genes listed in Supplementary Table 4 that encode proteins containing membrane-spanning domains. Genes are listed in decreasing order of statistical strength of differential expression (ANOVA).

| **Gene Name** | **Name in Figure 2** | **Fold**  **Change** | **ANOVA**  **p-value** |
| --- | --- | --- | --- |
| Guanylate binding protein 2, interferon-inducible | GBP2 | 1.30 | 0.0011 |
| Tetraspanin 18 | TSPAN18 | 2.09 | 0.0061 |
| Plexin domain containing 2 | PLXDC2 | 1.74 | 0.0080 |
| Seizure related 6 homolog (mouse)-like 2 | SEZ6L2 | 1.45 | 0.0080 |
| G protein-coupled receptor 125 | GPR125 | 1.32 | 0.0080 |
| Integral membrane protein 2C | ITM2C | 1.40 | 0.0081 |
| Leucine-rich repeats and immunoglobulin-like domains 3 | LRIG3 | 1.43 | 0.0128 |
| Calcitonin receptor-like | CALCRL | 1.53 | 0.0142 |
| Platelet derived growth factor D | PDGFD | 2.07 | 0.0152 |
| Doublecortin-like kinase 1 | DCLK1 | 1.55 | 0.0163 |
| Fibroblast growth factor receptor 2 | FGFR2 | 1.61 | 0.0165 |
| Stathmin-like 2 | STMN2 | 2.31 | 0.0167 |
| Gamma-glutamyltransferase 5 | GGT5 | 1.33 | 0.0188 |
| Collectin sub-family member 12 | COLEC12 | 1.67 | 0.0216 |
| Potassium channel, subfamily T, member 2 | KCNT2 | 1.44 | 0.0220 |
| Contactin associated protein-like 2 | CNTNAP2 | 1.52 | 0.0244 |
| Asporin | ASPN | 1.85 | 0.0254 |
| Carboxypeptidase E | CPE | 1.32 | 0.0265 |
| Prostaglandin F receptor (FP) | PTGFR | 1.70 | 0.0274 |
| CKLF-like MARVEL transmembrane domain containing 8 | CMTM8 | 1.43 | 0.0303 |
| Interferon induced transmembrane protein 1 (9-27) | IFITM1 | 1.49 | 0.0316 |
| Receptor tyrosine kinase-like orphan receptor 2 | ROR2 | 1.30 | 0.0347 |
| Glucoside xylosyltransferase 2 | GXYLT2 | 1.38 | 0.0415 |
| Sema domain, immunoglobulin domain (Ig), short basic domain, secreted, (semaphorin) 3A | SEMA3A | 1.44 | 0.0435 |
